# A Dynamic Co-expression Map of Early Inflorescence Development in *Setaria viridis* Provides a Resource for Gene Discovery and Comparative Genomics

**DOI:** 10.3389/fpls.2018.01309

**Published:** 2018-09-12

**Authors:** Chuanmei Zhu, Jiani Yang, Mathew S. Box, Elizabeth A. Kellogg, Andrea L. Eveland

**Affiliations:** Donald Danforth Plant Science Center, St. Louis, MO, United States

**Keywords:** inflorescence development, *Setaria*, panicoid grasses, transcriptome profiling, meristem, spikelet

## Abstract

The morphological and functional diversity of plant form is governed by dynamic gene regulatory networks. In cereal crops, grain and/or pollen-bearing inflorescences exhibit vast architectural diversity and developmental complexity, yet the underlying genetic framework is only partly known. *Setaria viridi*s is a small, rapidly growing grass species in the subfamily Panicoideae, a group that includes economically important cereal crops such as maize and sorghum. The *S. viridis* inflorescence displays complex branching patterns, but its early development is similar to that of other panicoid grasses, and thus is an ideal model for studying inflorescence architecture. Here we report a detailed transcriptional resource that captures dynamic transitions across six sequential stages of *S. viridis* inflorescence development, from reproductive onset to floral organ differentiation. Co-expression analyses identified stage-specific signatures of development, which include homologs of previously known developmental genes from maize and rice, suites of transcription factors and gene family members, and genes of unknown function. This spatiotemporal co-expression map and associated analyses provide a foundation for gene discovery in *S. viridis* inflorescence development, and a comparative model for exploring related architectural features in agronomically important cereals.

## Introduction

Extensive morphological diversity is exhibited by inflorescences across grass species, family Poaceae. Since the inflorescences bear grain, understanding the genetic and molecular bases for this variation can accelerate the generation of higher yielding crops through breeding or precision engineering. Grasses encompass the world’s important cereal crops; e.g., maize, sorghum, and millets in the subfamily Panicoideae, wheat, oats, and barley in subfamily Pooideae, and rice in subfamily Oryzoideae (Soreng et al., 2017). Many genes that regulate aspects of inflorescence architecture in grasses have been discovered and characterized, largely in maize and rice (Zhang and Yuan, 2014; Bommert and Whipple, 2017). In many cases gene function is conserved across species (Whipple et al., 2010; Bommert and Whipple, 2017), whereas in others, it varies between species or clades (McSteen, 2006; Whipple, 2017; Bommert and Whipple, 2017). Relatively little is known about how these genes interact in the larger context of a developmental network to control inflorescence form and how these networks are rewired across species.

All inflorescence structures are ultimately derived from a group of pluripotent cells called the inflorescence meristem (IM). The IM is indeterminate and transitions from the shoot apical meristem (SAM) during the shift from vegetative to reproductive growth (Bartlett and Thompson, 2014; Kyozuka et al., 2014). In grasses, the terminal reproductive structure is the spikelet, which bears one or more flowers that produce the seeds. The different routes that the IM takes to form a determinate spikelet meristem (SM) largely determine the morphology of the mature inflorescence (Kyozuka et al., 2014; Whipple, 2017). For example, the IM can produce SMs directly on its flanks, as in wheat, or it can produce few to many indeterminate branch meristems (BMs), which is typical for most grasses. Like the IM, BMs can produce SMs directly, as in finger millet, or can initiate higher order branches, as in rice. The IM and BM may ultimately convert to a terminal SM or simply cease development. Eventually, SMs initiate sterile bracts (glumes) and floral meristems (FMs), which produce lateral organs that differentiate into floral structures including lemma, palea, anthers, and ovary.

In species of the Andropogoneae tribe, which includes maize and sorghum, spikelets are borne in pairs where two SMs arise from a spikelet pair meristem (SPM). Paired spikelets also arose independently in some other closely related groups. In the “bristle clade” of grasses, which includes *Setaria* species and other members of the subtribe Cenchrinae (see Doust and Kellogg, 2002 and references therein), BMs can alternatively differentiate into sterile branches called bristles. While spikelets are not paired in these species, morphological analyses suggest that spikelets may be paired with bristles (Doust and Kellogg, 2002). Recent work in *Setaria viridis* showed that BMs poised to form bristles first initiate an SM identity program before the homeotic shift to bristle formation. This developmental switch is dependent on proper spatiotemporal synthesis of growth-promoting brassinosteroids (BRs) during SM development (Yang et al., 2017).

Despite this variation in inflorescence morphology among grasses, the core underlying developmental processes are shared, and often leverage common regulatory modules. Pathways that regulate IM size (Somssich et al., 2016), SM identity (Bommert and Whipple, 2017), and flower development (Hirano et al., 2014), for instance, are largely conserved across those grass species studied. However, these processes can vary by the spatiotemporal expression of certain factors, functional divergence of gene family members, and/or co-option of novel factors all together. For example, the RAMOSA1 (RA1) transcription factor (TF) is expressed at the base of SPMs in Andropogoneae species and acts to suppress branching by conferring determinacy on the SPM. The timing and degree of RA1 expression dictates inflorescence branching patterns; i.e., during maize tassel development *ra1* is induced early and suppresses higher order branching whereas in sorghum and *Miscanthus*, delayed *ra1* expression leads to highly branched inflorescences (Vollbrecht et al., 2005). By leveraging comparative transcriptome analyses across grasses, we can gain invaluable insight into the core components that regulate these processes, and what variations are associated with species-specific morphologies.

Transcriptome studies of early inflorescence development have been reported for a few important cereal crops, including maize (Eveland et al., 2014), rice (Furutani et al., 2006; Harrop et al., 2016), barley (Digel et al., 2015), and wheat (Feng et al., 2017), leading to systematic discovery of underlying regulatory modules. To our knowledge, maize is the only panicoid grass with existing transcriptome data across early inflorescence development (Eveland et al., 2014). Given the variation in architectures among important cereals in the Panicoideae, comparative expression maps from additional species will help determine conserved and species-specific components. Here, we present a comprehensive developmental and transcriptomics analysis of inflorescence development in *S. viridis*, a model panicoid grass with a rapid life cycle, sequenced genome, and emerging genetics and genomics toolkit (Doust et al., 2009; Brutnell et al., 2010; Bennetzen et al., 2012; Huang et al., 2016; Zhu et al., 2017). We profiled six sequential stages of inflorescence development that captured key events from the reproductive transition to floral organ differentiation and identified stage-specific co-expression signatures. Our detailed characterization of *S. viridis* inflorescence development at both morphological and molecular levels provides an invaluable resource for the community as a foundation for gene discovery and a comparative platform for studying diverse architectures in agronomically important cereal crops.

## Materials and Methods

### Plant Growth Conditions

The reference *S. viridis* genotype A10.1 was used for this study. A10.1 seeds from 2-year-old stocks were used to ensure full loss of dormancy and promote synchronized germination. Seeds were planted in MetroMix 360 soil (Sun Gro Horticulture company) and grown in a controlled growth chamber with the following conditions: temperature of 31°C/23°C (day/night), light intensity of 200 μmol/sq.meter/s for 12 h (6am–6pm) and 50% relative humidity, at the Donald Danforth Plant Science Center’s Integrated Plant Growth Facility. Plants were fertilized with Jack’s 15-16-17 (Hummert International) twice a week.

### Scanning Electron Microscopy

In our controlled conditions, the transition from a vegetative to IM occurred after 8 days after sowing (DAS) and before 10 DAS. Accordingly, vegetative SAMs were hand-dissected from plants at 8 DAS, and IMs from plants at 10, 11, 12, 13, 14, 15, 16, and 18 DAS. Samples were fixed immediately in FAA solution (3.75% formaldehyde, 50% ethanol, and 5% glacial acetic acid) and left overnight. They were then dehydrated using 50% ethanol for a minimum of 1 h and then shifted to 70% ethanol. For critical point drying, samples were dehydrated in an ethanol series (80, 95, 100, 100% ethanol) for at least 1 h for each step; they were then left in 100% ethanol overnight and then moved to fresh 100% ethanol from a newly opened bottle the next day. Samples were then critical point dried using a Tousimis Samdri-780a, mounted on stubs and sputter coated using a Tousimis Samsputter-2a. Images were taken with a Hitachi S2600 SEM at 20 kV at Washington University’s Central Institute for the Deaf.

### RNA Extraction, RNA-seq Library Construction, Sequencing, and Analysis

Inflorescences were hand-dissected into fresh 100% acetone on dry ice from *S. viridis* seedlings at 10, 12, 14, 15, 16, and 18 DAS. Depending on the representative size at a given stage, 10–30 individual inflorescence primordia were pooled per biological replicate; three to four biological replicates were collected for each stage. All sampling was performed within a 2-h window in the morning to control for circadian effects. Acetone was removed and samples were flash-frozen in liquid nitrogen and ground into a fine powder using 3 mm tungsten-carbide beads (Qiagen) in a Tissue Lyser-II. Total RNA was isolated using the PicoPure RNA Isolation Kit (Thermo Fisher Scientific) with in-column DNase I treatment following manufacturer’s protocols. RNA-seq libraries were generated from 1 μg total RNA using the NEBNext Ultra Directional RNA Library Prep Kit for Illumina (New England BioLabs Inc.) and size-selected for 200 bp inserts. Libraries were quantified on a Bioanalyzer (Agilent 2100) using a DNA 1000 chip to confirm the insert size and quantified again on a Qubit 3.0 Fluorometer (Life Technologies) to ensure different libraries were equally loaded, and then sequenced using standard Illumina protocols (Illumina, Inc.) for either paired-end (PE) or single-end (SE) sequencing at 100 bp on the Illumina HiSeq 2500 platform at the University of Illinois at Urbana-Champaign W.M. Keck Center.

Sequenced reads were quality-checked using fastQC with Phred scores all above 30. For read mapping and transcript quantification, we used *Salmon* (v0.8.1) (Patro et al., 2017) in a quasi-mapping mode for indexing reference transcripts from the *S. viridis* primary transcript file (Sviridis_311_v1; Phytozome^1^ v12.1) (**Supplementary Table S1**). Transcripts were quantified using the option numBootstraps 30. Parameters not specified were run as default. *Salmon* outputs were imported into R with the Bioconductor package *tximport* (v1.0.3). Raw counts and transcripts per million (TPM) were extracted for 35,214 *S. viridis* primary transcripts (**Supplementary Table S2**). All subsequent analyses were conducted using TPM (Soneson et al., 2015).

### Gene Co-expression Analyses

To reduce noise from genes with ubiquitously low expression or that do not change expression during development, we applied a set of criteria to filter genes that: (1) showed more than twofold change in expression between at least two of the stages, and (2) collectively had an expression value of at least 1 TPM across all stages. This resulted in 11,425 dynamically expressed genes during inflorescence development (**Supplementary Table S3**). To determine the optimal number of clusters to use for fuzzy c-means (FCM) cluster analysis, we used two functions in the R Bioconductor package, *Mfuzz* (Kumar and Futschik, 2007): (1) Dmin, which calculates the minimum centroid distance for a range of cluster numbers and (2) cselection, which reports the cluster number where empty clusters are detected in the repeated soft clustering. Cluster number was chosen for FCM analysis based on the centroids of each cluster being well separated based on Dmin and there were no empty clusters based on cselection. FCM was performed using *Mfuzz*. The 11,425 genes were clustered into groups based on their standardized mean expression profiles (for each transcript, average expression is 0 and the standard deviation across 6 stages is 1). Each gene was assigned to the cluster with its highest membership score. Heatmaps and hierarchical clustering using Euclidean distances and complete linkage clusters were generated using MeV^2^ (version 4.8) (Saeed et al., 2003).

### Gene Annotation and Homology Searches

The *S. viridis* genome (v1.1) and annotation files were downloaded from Phytozome v12.1. Functional annotations were assigned to *S. viridis* genes based on these files and information extracted from EnsemblPlants Biomart^3^. To determine homologs of *S. viridis* genes in other grass species [maize (v3 and v4 genomes), sorghum (v3.1.1), *Brachypodium* (v3.1), and *Setaria italica* (v2.2)], blastp^4^ was used with two as the maximum target sequence for each gene. All genome sequence data were downloaded from Phytozome v12.1, except for maize v4, which was downloaded from MaizeGDB^5^.

### Phylogenetic Analysis of MADS-Box Family TFs

An HMM (Hidden Markov Model^6^, HMMER 3.1b2) was used to classify genes encoding MADS-Box proteins in the proteome database of *S. viridis* (v1.1) and coding sequences of the primary transcripts were retrieved from Phytozome^1^. Previously identified MIKC type MADS-box genes from Arabidopsis, rice and maize genomes (Parenicová et al., 2003; Arora et al., 2007; Zhao et al., 2011) were included in building the phylogenetic tree. All protein coding sequences were manually checked for the presence of a MADS domain and were aligned using MUSCLE (Edgar, 2004) to construct a maximum likelihood tree by RAxML (Edgar, 2004; Stamatakis, 2014), with 1,000 bootstrap replicates. The final trees were drawn with the R Bioconductor package, *phytools* (Revell, 2011).

### Weighted Gene Co-expression Network Analysis

The R Bioconductor package *wgcna* was used to perform weighted gene co-expression network analysis (WGCNA) on the same set of 11,425 genes used in FCM analysis (Langfelder and Horvath, 2008). A matrix of all genes with their TPM values across 23 samples (including individual biological replicates) was used as input. An adjacency matrix was generated to determine similarity between genes (i.e., correlation for every gene pair across the 23 samples) and transformed through a soft-thresholding procedure using the function “pickSoftThreshold,” where a soft power of 18 was chosen for module detection. A topological overlap measure (TOM) was then calculated from the adjacency matrix to estimate network interconnectedness. The dissimilarity of TOM (1-TOM) was used as the input for average linkage hierarchical clustering to identify co-expression modules. Module eigengenes (MEs), the first principal component of a given module, was used as a representative gene expression profile for that module. Modules were further merged based on their MEs (using cutHeight = 0.25) and the module membership (MM) for each gene indicated the degree of similarity between its expression profile and each ME. The entire network including WGCNA-calculated weights for each edge between genes (nodes) is available on NCBI GEO. Gene-to-gene connections were filtered if the weight of interaction was <0.02. Sub-networks were visualized using Cytoscape^7^ v3.4.10.

## Results and Discussion

### Morphological Characterization of Inflorescence Development in *S. viridis*

**FIGURE 1:**
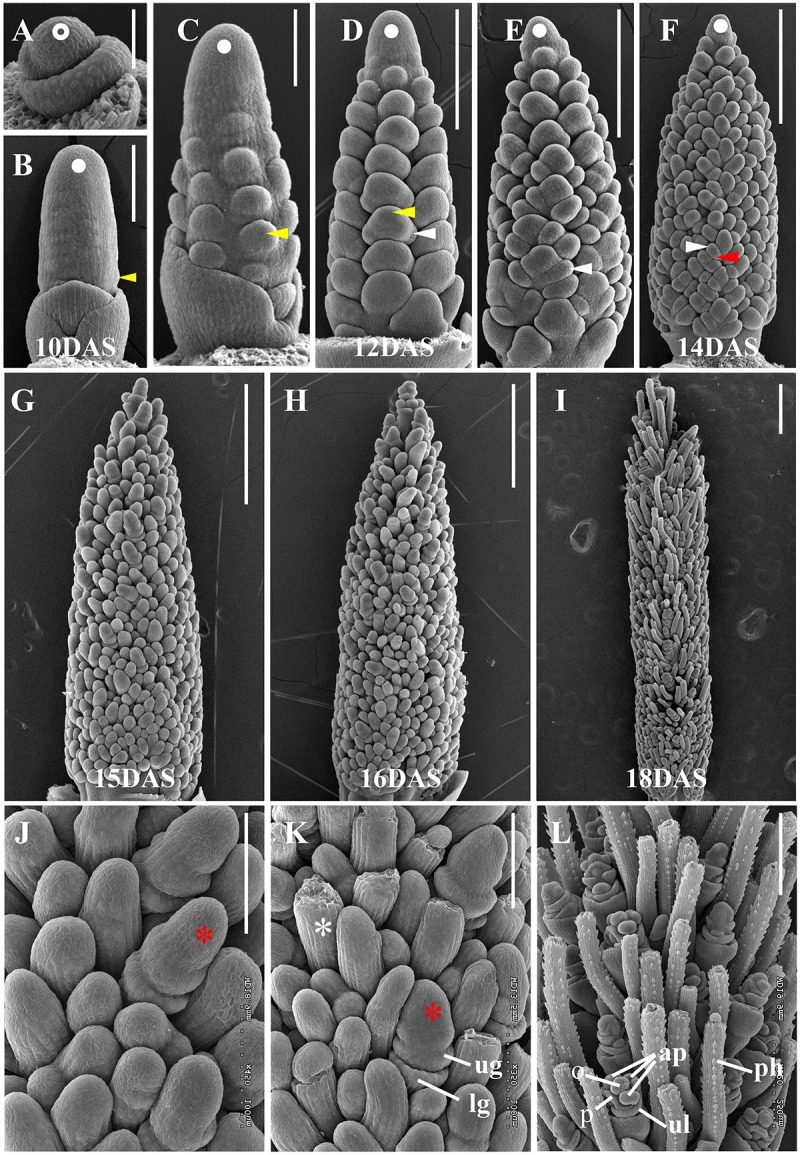
Key developmental stages of *Setaria viridis* inflorescence development by SEM. **(A)** At 8 DAS, the *S. viridis* SAM (*white open circle*) had not yet transitioned to reproductive development. **(B)** By 10 DAS, the SAM had transitioned to an indeterminate IM (*white dot*) and primary BMs (*yellow arrow*) were initiated. As development proceeds through **(C)** 11 DAS, **(D)** 12 DAS, **(E)** 13 DAS, and **(F)** 14 DAS stages, primary, secondary, and higher order branching was progressively initiated (white dot, IM; yellow arrow, primary BM; white arrow, secondary BM; red arrow, tertiary BM). **(G**, **J)** At 15 DAS, SMs (*red asterisk*) had begun to differentiate and by 16 DAS **(H**, **K)**, were distinguishable from developing bristles (*white asterisk*) and initiated glume primordia (lg, lower glume; ug, upper glume). **(I**, **L)** At 18 DAS, bristles were elongated and developed prickle hairs (ph) and SMs further differentiated floral organs (ul, upper lemma; p, palea; ap, anther primordium; o, ovary). Developmental stages that were used for RNAseq-based transcript profiling are labeled. Scale bars = 50 μm in **(A)**, 100 μm in **(B**, **C**, **J**, and **K)**, 250 μm in **(D**–**F** and **L)**, and 500 μm in **(G**–**I)**.

To characterize the developmental progression of the *S. viridis* inflorescence, we used scanning electron microscopy (SEM) to examine sequential stages from floral transition to floral organ development. At 8 DAS, the SAM had not yet transitioned, but had emerged from surrounding leaf primordia (**Figure 1A**). By 10 DAS, the SAM had transitioned to the IM and BMs were initiated but barely visible (**Figure 1B**). By 11 DAS, primary BMs had initiated at the base of the developing inflorescence and continued to form in a helical pattern at 12 DAS (**Figures 1C,D**). From 12 to 14 DAS, secondary and tertiary BMs developed sequentially in a distichous pattern (**Figures 1D–F**). By 15 DAS, the IM had ceased to produce new BMs and those at the inflorescence tip were the first to differentiate into SMs or bristles (**Figures 1G,J**). At 16 DAS, differentiation of SMs and bristles continued basipetally and the two developing structures became morphologically distinguishable. SMs then initiated glumes and FMs, while bristles started to elongate and form an indented ring below the meristem tip (**Figures 1H,K**), which often later detached (Doust and Kellogg, 2002; Yang et al., 2017). By 18 DAS, floral organs such as lemma, palea, and anther primordia formed in the spikelets, and bristles further elongated and produced prickle hairs (Doust and Kellogg, 2002; **Figures 1I,L**).

### Transcriptome Profiling and Stage-Specific Expression of TF Families

To establish a dynamic transcriptome map of *S. viridis* inflorescence development, we used RNA-seq to link global changes in gene expression with developmental transitions. Based on our detailed morphological characterization, we selected six stages that captured key events in inflorescence development for transcriptome profiling by RNA-seq: the initiation of the IM (10 DAS), primary and secondary (12 DAS), and higher order (14 DAS) branching events, transition to SMs (15 DAS), differentiation of spikelets and bristles (16 DAS), and development of floral organs (18 DAS) (**Figure 1**). For each of these six stages, inflorescence primordia were hand-dissected and RNA-seq libraries prepared for three to four biological replicates as described in the Section “Materials and Methods” (**Supplementary Table S1**). Transcript abundance was quantified in TPM at each of the six stages; these analyses showed strong correlations among biological replicates and dynamic patterns of gene expression across developmental stages (**Supplementary Figures S1**, **S2** and **Supplementary Table S2**). Based on our filtering criteria described in the section “Materials and Methods,” all analyses in this manuscript were performed using a subset of 11,425 dynamically expressed genes (**Supplementary Table S3**).

**FIGURE 2:**
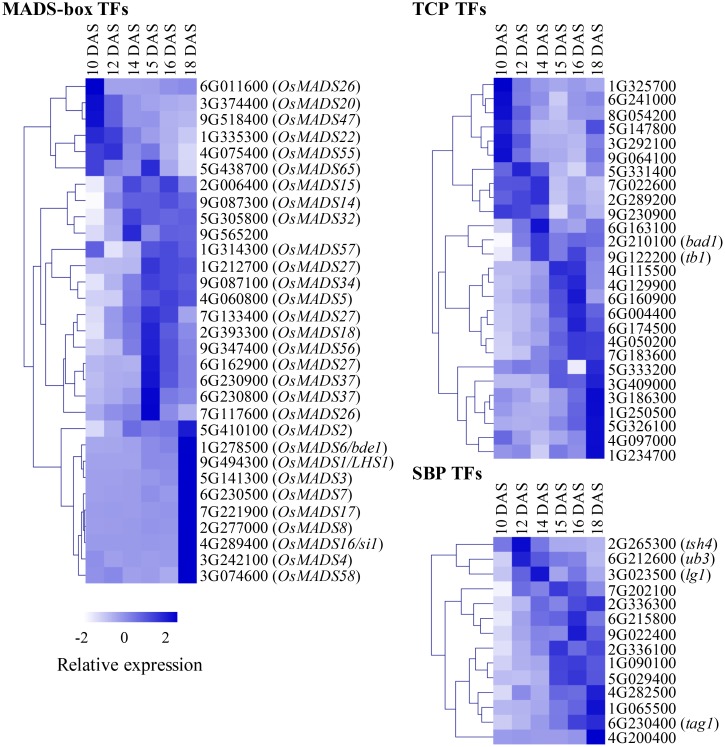
Transcription factor (TF) family members show dynamic expression profiles across inflorescence development. Individual members of MADS-box, TCP and SBP TF families were annotated, TPM values for each were normalized based on a Z-scale and plotted as a heatmap across the six stages of *Setaria viridis* inflorescence development. For MADS-box genes, names of orthologous genes from rice are included. For TCP and SBP families, orthologs of classical maize genes are indicated. *White-to-dark* shading represents low-to-high relative expression level.

We explored developmental dynamics of TF families across the six stages. Spatiotemporal expression and combinatorial action of TF family members fine-tune developmental decisions, and knowledge of their individual expression profiles can provide insight into underlying regulatory mechanisms. We annotated several TF families in *S. viridis* based on annotations of functional TF domains from various sources. Specifically, we focused on families with multiple members previously implicated in plant development and plotted relative expression of family members across the six stages: MADS-box, TCP, SBP (**Figure 2**), AP2/ERF, bHLH, C2H2_Zinc, bZIP, LOB, and NAC families (**Supplementary Figure S3**; see abbreviations in **Box 1**). Individual TF family members showed dynamic expression profiles during early inflorescence development in *S. viridis* (**Figures 2**, **Supplementary Figures S3**, **S4** and **Supplementary Table S4**). These included orthologs of previously characterized developmental regulators from maize and rice, as well as many uncharacterized family members with potential functions in inflorescence development based on their co-expression patterns. For example, we identified a set of 21 candidate TFs that were highly expressed and showed large fold changes in expression that peaked at specific stages of inflorescence development (**Supplementary Figure S4** and **Supplementary Table S4**).

**Box 1:** Abbreviations and names of TF families annotated in this study.

	Transcription factor (TF) family
AP2/ERF	AP2/ethylene-responsive factor
bHLH	Basic helix-loop-helix
bZIP	Basic-leucine zipper protein
C2H2_Zinc	C2H2-zinc finger protein
LOB	Lateral organ boundary domain proteins
NAC	NAM, ATAF1/2, and CUC2 domain proteins
SBP	Squamosa promoter-binding protein
TCP	Teosinte branched 1/CYCLOIDEA/PROLIFERATING CELL FACTOR1

In addition, we performed a detailed phylogenetic analysis for the MIKC type MADS-box family based on these annotations to determine homology between the *S. viridis* genes and those in maize, rice and/or Arabidopsis (**Supplementary Figure S5**). This phylogenetic framework enables comparative analyses among MADS-box family members that have been shown in multiple systems to work in a combinatorial manner to regulate development (Theißen et al., 2016; Bartlett, 2017).

### Modules of Co-expressed Genes During *S. viridis* Inflorescence Development

To define signatures of gene expression across this developmental trajectory, we used an FCM clustering approach (Kumar and Futschik, 2007) and our filtered set of 11,425 dynamically expressed genes. Based on simulations and selection criteria as described in the Section “Materials and Methods,” we determined 25 as an optimal number of clusters to use for the FCM analyses (**Supplementary Figure S6A**). FCM was used to assign a membership score for each gene to each cluster, and based on the highest score for each gene, 25 clusters with unique expression profiles were defined ranging from 290 to 608 genes per cluster (**Supplementary Figure S6B** and **Supplementary Table S3**).

**FIGURE 3:**
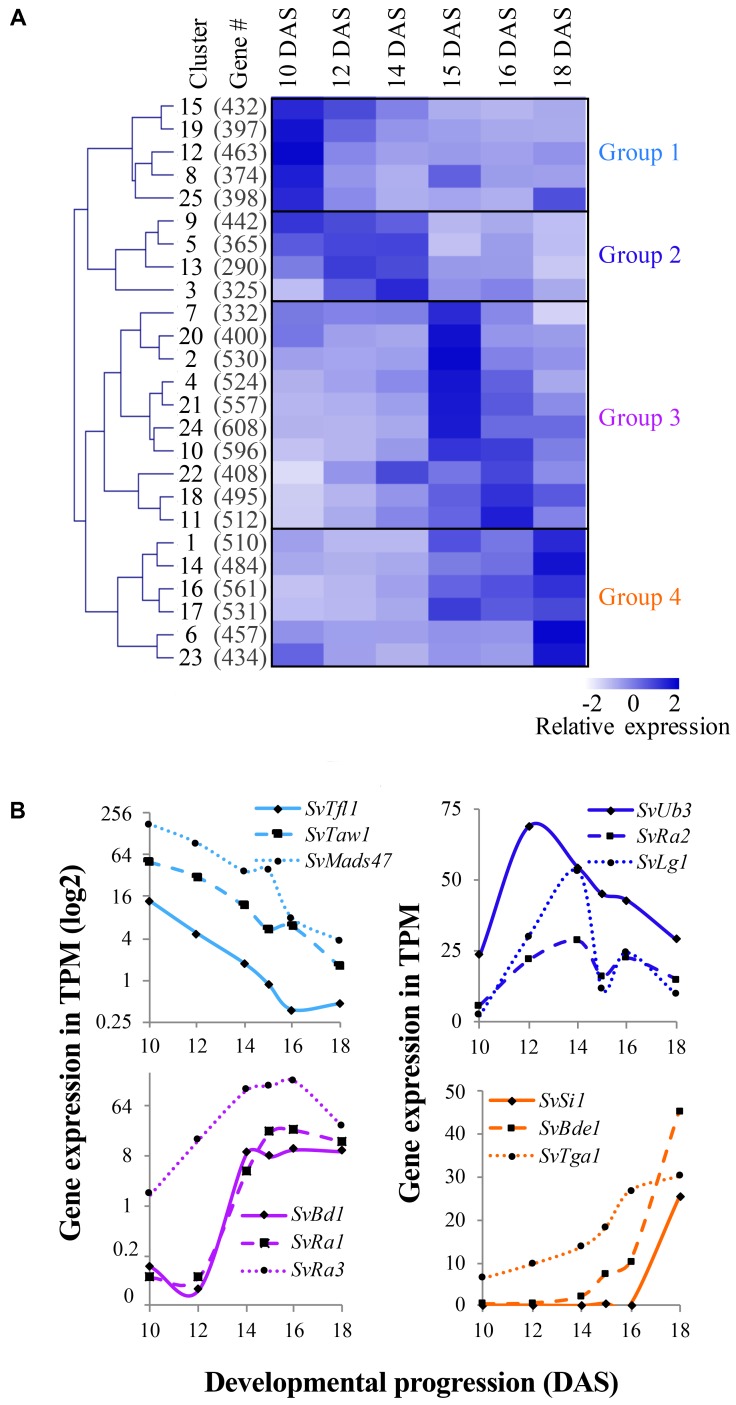
Co-expression analysis revealed distinct gene expression signatures during *Setaria viridis* inflorescence development. **(A)** FCM clustering of 11,425 dynamically expressed genes identified 25 co-expression clusters. The mean relative expression value of genes in each cluster across development was used for hierarchical clustering and is displayed by the heatmap. Overall clusters organized into four general groups of expression associated with key developmental transitions. *White-to-dark* shading represents low-to-high relative expression level. **(B)** Within each of the four expression groups, homologs of known developmental genes from other species showed expected expression patterns.

The mean relative expression value across the six developmental stages was determined for each of the 25 clusters and hierarchical clustering on these values revealed four groups (**Figure 3A**), each representing a distinct developmental expression pattern: (1) floral transition and IM identity, (2) BM initiation and determinacy, (3) SM differentiation and FM initiation, and (4) floral organ development. We first located orthologs of known developmental genes; expression profiles of representative genes within each group were comparable to those of their orthologs in maize and rice, consistent with their predicted roles in development (**Figure 3B** and **Supplementary Table S3**). In the following four subsections, we describe expression of these and other key developmental regulators during *S. viridis* inflorescence development compared to other grasses and highlight some key findings from each of the major expression groups. For simplicity, we listed full names and abbreviations of genes discussed in **Table 1**.

**Table 1:** *Setaria viridis* genes, short name descriptions and homologs in maize, rice, and Arabidopsis.

*S. viridis*		Maize	Rice	Arabidopsis
Sevir.8G033800	*SvTfl1*	*Zea centroradialis*	*RICE CENTRORADIALIS*	*TERMINAL FLOWER 1*
Sevir.5G394700	*SvLg2*	*Liguleless 2*		
Sevir.9G221800	*SvTaw1*		*TAWAWA 1*	
Sevir.4G229000	*SvMoc1*		*MONOCULM 1*	
Sevir.3G028500	*SvWus*		*TILLERS ABSENT 1*	*WUSCHEL*
Sevir.7G234000	*SvLfy*	*Zea floricaula*	*ABERRANT PANICLE ORGANIZATION 2*	*LEAFY*
Sevir.6G212600	*SvUb3*	*Unbranched 3*	*WEALTHY FARMER’S PANICLE*	
Sevir.2G265300	*SvTsh4*	*Tassel shealth 4*		
Sevir.3G136200	*SvTsh1*	*Tassel sheath 1*	*NECK LEAF 1*	*HANABA TARANU*
Sevir.3G023500	*SvLg1*	*Liguleless 1*		
Sevir.5G116100	*SvRa2*	*Ramosa 2*		
Sevir.4G025200	*SvBaf1*	*Barren stalk fastigiate 1*	*DEPRESSED PALEA 1*	*AT-HOOK NUCLEAR LOCALIZED 22*
Sevir.5G116300	*SvVt2*	*Vanishing tassel 2*		
Sevir.3G410700	*SvBif2*	*Barren inflorescence 2*		
Sevir.2G302300	*SvDfl1*	*Delayed flower 1*		
Sevir.9G259300	*SvPla1*			*PLASTOCHRON 1*
Sevir.5G251100	*SvSpi1*	*Sparse inflorescence 1*		
Sevir.5G374100	*SvBa1*	*Barren stalk 1*	*LAX PANICLE 1*	
Sevir.2G437800	*SvBd1*	*Branched silkless 1*	*FRIZZY PANICLE*	
Sevir.2G209800	*SvRa1*	*Ramosa 1*		
Sevir.2G407500	*SvRa3*	*Ramosa 3*		
Sevir.4G119100	*SvFea4*	*Fasciated ear 4*		*PERIANTHIA*
Sevir.4G294000	*SvTd1*	*Thick tassel dwarf 1*		*CLAVATA 1*
Sevir.8G183800	*SvFon2*		*FLORAL ORGAN NUMBER 2*	*CLAVATA 3*
Sevir.9G107600	*SvKn1*	*Knotted 1*		*BREVIPEDICELLUS*
Sevir.2G029800	*SvRs1*	*Rough sheath 1*		
Sevir.2G237500	*SvBrm*			*BRAHMA*
Sevir.4G112300	*SvSyd*			*SPLAYED*
Sevir.4G124400	*SvCuc2*			*CUP-SHAPED COTYLEDON 2/3*
Sevir.6G213600	*SvCuc3*			
Sevir.2G210100	*SvBad1*	*Branch angle defective 1/wavy auricle in blade 1*	*RETARDED PALEA 1*	
Sevir.9G122200	*SvTb1*	*Teosinte branched 1*		
Sevir.4G289400	*SvSi1*	*Silky 1*	*SUPERWOMAN1*	
Sevir.1G278500	*SvBde1*	*Bearded ear 1*		
Sevir.9G494300	*SvLhs1*		*LEAFY HULL STERILE1*	
Sevir.6G230400	*SvTga1*	*Teosinte glume architecture 1*		
Sevir.5G086100	*SvSl1*		*STAMENLESS 1*	
Sevir.1G255900	*SvTob1*	*Yabby 15*	*TONGARI-BOUSHI 1*	
Sevir.9G265300	*SvAn1*	*Anther ear 1*		
Sevir.9G439800	*SvTs2*	*Tassel seed 2*		

### Group 1: Floral Transition and IM Identity

Genes in Group 1 (including clusters 8, 12, 15, 19, and 25) showed highest expression at 10 DAS as the IM was initiated and decreased at later stages of development (**Figure 3A**, **Supplementary Figure S6** and **Supplementary Table S3**). We inferred that genes involved in regulating the transition to reproductive growth, IM identity, and/or primary branch initiation would be included in these clusters. For example, *SvTfl1* (Sevir.8G033800), an ortholog of *TERMINAL FLOWER1*, a general repressor of flowering in many species including maize, rice, and Arabidopsis (Nakagawa et al., 2002; Danilevskaya et al., 2010; Hanano and Goto, 2011), was grouped in cluster 19 (**Figure 3B**). Three other *TFL1*-like homologs clustered in Group 1; Sevir.7G334800, a closely related paralog of *SvTfl1*, Sevir.7G097700 and Sevir.1G183200. The latter gene, which we named *SvTfl1-like*, was expressed markedly higher than the other two at 10–12 DAS in cluster 15 (**Supplementary Table S3**). Co-expressed in cluster 15 was *SvLg2* (Sevir.5G394700), the ortholog of *liguleless 2* (*lg2*) from maize, which encodes a bZIP TF responsible for early establishment of lateral organ boundary positioning (Harper and Freeling, 1996; Walsh et al., 1998). Loss-of-function *lg2* mutants in maize produce few to no tassel branches. Consistent with its expression profile in *S. viridis* (**Supplementary Figure S4**), *lg2* in maize has been implicated in the transition from vegetative to reproductive growth (Walsh and Freeling, 1999).

**FIGURE 4:**
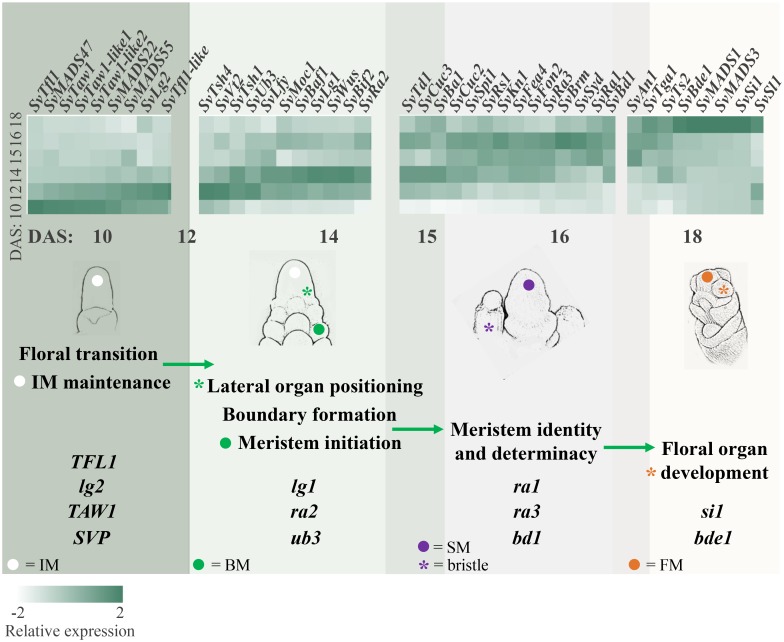
Orthologs of genes known to regulate inflorescence architecture in maize and rice showed expected expression patterns during *Setaria viridis* inflorescence development and transitions between meristem types. The schematic diagrams sequential developmental transitions captured by the RNA-seq data and shows relative expression of genes predicted to control these transitions. For example, consistent with the initiation of BMs from the IM between 10 and 14 DAS, positive regulators of lateral meristem formation (e.g., *SvBif2* and *SvUb3)* showed increased expression between 10 and 14 DAS, and negative regulators of axillary meristem formation (e.g., *SvTd1* and *SvFea4*) increased later at 14–16 DAS. Genes displayed in the heatmap are divided by group and developmental processes can transition across groups. *Light-to-dark* color indicates low-to-high relative expression.

In rice, *TAWAWA1* (*TAW1*) encodes a nuclear protein belonging to the *ALOG* (*ARABIDOPSIS LSH1 and ORYZA G1*) gene family that is highly expressed in the IM and BMs early in inflorescence development, and decreases during the phase change to SM identity (Yoshida et al., 2013). *TAW1* promotes indeterminate IM and BM activity by suppressing acquisition of SM identity, and thus is a major regulator of inflorescence architecture (Yoshida et al., 2013). The *S. viridis* ortholog, *SvTaw1* (Sevir.9G221800), was found in cluster 19 (**Figures 3A,B**), which shows a very similar expression profile to cluster 15, consistent with *TAW1* expression in rice. Five other *ALOG* genes were found in Group 1 and shared similar expression profiles (Table S3); Sevir.7G17600 and Sevir.1G244200, which we named *SvTaw1-like 1* and *SvTaw1-like 2*, respectively, shared highest homology with *SvTaw1* and were co-expressed in cluster 15 (**Figure 4**). Functional studies of other *ALOG* genes in various species indicate that they generally share a role in developmental phase changes and organ identity, and their spatiotemporal expression patterns determine their specificity (Yoshida et al., 2009; Cho and Zambryski, 2011; Takeda et al., 2011). Notably, three genes belonging to a subfamily of MADS-box TFs called *SHORT VEGETATIVE PHASE* (*SVP*), were also co-expressed in Group 1 (**Figures 3B**, **4** and **Supplementary Table S3**). In rice, homologs of these *SVP* genes (*OsMADS47*, *OsMADS55*, and *OsMADS22*) suppress FM initiation and were positively regulated by *TAW1* to promote indeterminacy (Yoshida et al., 2013; Kyozuka et al., 2014).

### Group 2: BM Initiation and Determinacy

Genes in Group 2 (clusters 3, 5, 9, and 13) tended to show highest expression at 12 and 14 DAS, decreasing by 15 DAS and into later stages (**Figure 3A** and **Supplementary Figure S6**). This expression pattern coincides with the initiation, maintenance and patterning of BMs, which occurs mostly between 10 and 14 DAS, and is largely completed by 15 DAS. Among genes in this group were those with putative roles in axillary meristem initiation and maintenance. For example, *SvMoc1* (Sevir.4G229000), a GRAS family protein homologous to *MONOCULM 1* (*MOC1*) from rice, was found in cluster 3. This expression pattern is consistent with a possible role for *SvMoc1* in axillary meristem initiation, analogous to how it controls tiller outgrowth in rice (Li et al., 2003). The rice *WUSCHEL* (*WUS*) ortholog, *TILLERS ABSENT 1* (*TAB1*/*OsWUS*), was also shown to play a role in initiating axillary meristems (Tanaka et al., 2015). Consistent with this, the *S. viridis* ortholog, *SvWus* (Sevir.3G028500), was co-expressed in cluster 3 (**Supplementary Table S3**). Also co-expressed with these developmental regulators was *SvLfy*, the *S. viridis* ortholog of *zea floricaula1/2/ABERRANT PANICLE ORGANIZATION 2 (APO2)/LEAFY* (*SvLfy*, Sevir.7G234000). These genes play universal roles in regulation of meristem determinacy, but with some species-specific differences; *LFY* and *zfl1/2* promote meristem determinacy and FM identity in Arabidopsis and maize, respectively (Schultz and Haughn, 1991; Bomblies et al., 2003), but *APO2* promotes meristem indeterminacy as well as BM initiation in rice (Kyozuka et al., 1998; Ikeda-Kawakatsu et al., 2012).

Among the SBP TFs annotated in developing *S. viridis* inflorescences (**Figure 2**), three were associated with Group 2. *SvUb3* (Sevir.6G212600), the ortholog of *unbranched 3 (ub3)* from maize and *WEALTHY FARMERS PANICLE (WFP)* from rice, was expressed in cluster 3. The function of this gene is generally conserved in both species in controlling partitioning of cells between lateral organ formation and meristem maintenance (Jiao et al., 2010; Miura et al., 2010; Chuck et al., 2014; Li et al., 2016). In maize, *ub3* shares redundant roles with *tassel sheath 4* (*tsh4*), another SPB TF that functions also to suppress formation of leaves in the inflorescence (Chuck et al., 2010, 2014). *SvTsh4* (Sevir.2G265300) was also found in Group 2, and was co-expressed in cluster 13 with *SvTsh1* (Sevir.3G136200), which encodes a GATA TF that functions in the same pathway (Wang et al., 2009; Whipple et al., 2010; **Figures 2**, **3A**).

Initiation of lateral organs involves the formation of boundaries, regions of decreased cell division that separate the growing organ primordia from meristematic signals to promote proper organ identity. Genes implicated in setting up lateral organ boundaries were identified in Group 2. *SvLg1* (Sevir.3G023500), the third SBP TF in this group and orthologous to *liguleless 1* (*lg1*) from maize (Moreno et al., 1997; Lewis et al., 2014) and *OsLG1* from rice (Lee et al., 2007), was found in cluster 3 (**Figures 2**, **3B**). In maize, *lg1* is classically known for its role in setting up the proximal-distal boundary that separates leaf blade from sheath and, along with *lg2*, is required for ligule and auricle development, thus controlling leaf angle. Recently, *lg1* was also shown to regulate inflorescence architecture by controlling tassel branch number in maize (Lewis et al., 2014) and branch angle in rice (Ishii et al., 2013; Zhu et al., 2013). Consistent with our data from *S. viridis*, *lg2* was proposed to act upstream of *lg1* in maize, with *lg2* expression initiated earlier in development to specify boundary position (Harper and Freeling, 1996; Lewis and Hake, 2016).

Also co-expressed with *lg1* in cluster 3 was *SvRa2* (Sevir.5G116100), ortholog of the maize *ramosa 2* (*ra2*) gene, which encodes a *LATERAL ORGAN BOUNDARY* (*LOB*) domain TF that is expressed transiently in positions where axillary meristems will form on the inflorescence (Moreno et al., 1997; Bortiri et al., 2006; **Figure 3B** and **Supplementary Figure S3**). In maize, *ra2* and *lg1* were shown to act in parallel pathways to control branch number and angle during early inflorescence development (Bai et al., 2012). The *S. viridis* ortholog of maize *barren stalk fastigiate 1* (*baf1*), *SvBaf1* (Sevir.4G025200), was also co-expressed in cluster 3 (**Figure 4** and **Supplementary Table S3**). In maize, *baf1* encodes an AT-hook protein that is involved in demarcating the boundary region of axillary meristems and plays a role in their initiation (Gallavotti et al., 2011).

The positioning of axillary meristems is strongly influenced by transport and function of the phytohormone auxin. As auxin flows in and out of emerging primordia, localized auxin response maxima arise at places of lateral meristem initiation, and this mechanism is generally conserved in maize inflorescence development (Barazesh and McSteen, 2008; Gallavotti, 2013; Eveland et al., 2014). Therefore, we would expect suites of auxin-related genes to be dynamically expressed between 12 and 15 DAS in *S. viridis* while branching occurs; i.e., in Groups 2 and 3. We annotated genes in these groups based on homology to those implicated in synthesis, transport, signaling and/or response of auxin, and plotted their expression profiles (**Supplementary Figure S7** and **Supplementary Table S5**). The expression of *SvVt2* (Sevir.5G116300), the ortholog of maize *vanishing tassel 2* (*vt2*), was initiated early in development and associated with cluster 13, while expression of *SvBif2* (Sevir.3G410700), the ortholog of *barren inflorescence 2* (*bif2*), peaked slightly later and was in cluster 3. *Vt2* encodes a tryptophan aminotransferase that regulates an early step of auxin biosynthesis (Phillips et al., 2011), and *bif2* encodes a PINOID serine/threonine kinase that regulates auxin transport (McSteen et al., 2007; **Supplementary Figure S7**).

Other key genes implicated in auxin-mediated lateral branching in maize and rice inflorescence development were expressed slightly later and in Group 3 (**Figure 3A** and **Supplementary Figure S7**). For example, genes in cluster 22 increased expression between 10 and 14 DAS, but expression was stable after 15 DAS unlike Group 2 clusters that decreased expression between 15 and 18 DAS (**Figure 3A** and **Supplementary Figure S6**). These included *S. viridis* orthologs of maize *sparse inflorescence 1* (*spi1*; *SvSpi1*; Sevir.5G251100), encoding a *YUCCA-like* gene involved in a late step of auxin biosynthesis (Gallavotti et al., 2008), and *barren stalk 1* (*ba1*)/*LAX PANICLE 1* (*LAX1*) (*SvBa1;* Sevir.5G374100), a bHLH protein involved in auxin signaling (Komatsu et al., 2003a; Gallavotti et al., 2004; **Supplementary Figure S7**). In maize, functional BA1 is required for creation of auxin maxima at the meristem anlagen to promote lateral meristem initiation. Our expression data from *S. viridis* are consistent with expression and genetics analyses in maize that show *ba1* acts downstream of *baf1* (Gallavotti et al., 2011).

### Group 3: SM Differentiation and FM Initiation

Clusters 10, 11, 18, and 22 in Group 3 have similar expression signatures that show a progressive increase during early stages of development, peaking around 16 DAS, and decreasing slightly by 18 DAS (**Figure 3A** and **Supplementary Figure S6**). Among these clusters were a number of genes related to axillary meristem identity and determinacy. For example, *SvBd1* (Sevir.2G437800), a marker of SM identity in *S. viridis* (Yang et al., 2017), was found in cluster 18 (**Figure 3B** and **Supplementary Table S3**). Orthologs of this AP2/ERF TF across grass species, e.g., *branched silkless 1 (bd1)* in maize, *FRIZZY PANICLE* (*FZP)* in rice, and *MORE SPIKELETS 1* (*MOS1*) in *Brachypodium distachyon*, share a conserved function in specifying SM identity (Chuck et al., 2002; Komatsu et al., 2003b; Derbyshire and Byrne, 2013).

Genes in the maize RAMOSA pathway regulate meristem determinacy prior to SM identity (Vollbrecht et al., 2005). Among these genes, *ra2* is widely conserved across grasses and its expression comes on early during axillary meristem initiation. Consistent with genetics and expression data from maize (Bortiri et al., 2006; Satoh-Nagasawa et al., 2006), the *S. viridis* orthologs of *ra1* (*SvRa1;* Sevir.2G209800) and *ramosa 3* (*ra3*) (*SvRa3*; Sevir.2G407500), which encodes a trehalose-phosphate phosphatase (Satoh-Nagasawa et al., 2006), were expressed after initiation of *SvRa2* and were found in clusters 10 and 11, respectively, their expression peaking at 15–16 DAS (**Figures 3A,B**).

Genetic interactions among genes controlling meristem determinacy and meristem size pathways in maize indicated these processes interface generally (Bommert and Whipple, 2017). Interestingly, many genes orthologous to known players in meristem size pathways were also co-expressed in Group 3. For example, *SvFea4* (Sevir.4G119100), the ortholog of *fasciated ear 4* (*fea4*) from maize and *PERIANTHIA (PAN)* from Arabidopsis encoding a bZIP TF that negatively regulates meristem size (Maier et al., 2009; Pautler et al., 2015), was found in cluster 22 (**Figure 3A** and **Supplementary Figure S3**). Regulatory components of the CLAVATA-WUSCHEL negative feedback signaling pathway central to meristem maintenance (Somssich et al., 2016) were also co-expressed in cluster 22. Among these were *SvTd1* (Sevir.4G294000) and *SvFon2* (Sevir.8G183800), orthologs of maize *thick tassel dwarf 1 (td1)/CLAVATA1*, encoding a leucine-rich repeat receptor-like kinase (Bommert et al., 2005), and rice *FLORAL ORGAN NUMBER 2/CLV3*, encoding a CLV3/ESR related peptide (Chu et al., 2006; Goad et al., 2017), respectively (**Supplementary Figure S2** and **Supplementary Table S3**). Both *td1/CLV1* and *FON2/CLV3* negatively control meristem size.

Certain Class I KNOX homeodomain TFs also function in pathways to maintain meristem cell identity and size. Orthologs of the maize *knotted 1 (kn1)* and *rough sheath 1* (*rs1*) genes, *SvKn1* (Sevir.9G107600) and *SvRs1* (Sevir.2G029800), respectively, both homologs of the Arabidopsis *BREVIPEDICELLUS* (*BP*) gene, were co-expressed in cluster 11 (**Figure 3A** and **Supplementary Figure S6**). In maize, *kn1* is a key marker of meristem maintenance (Kerstetter et al., 1997; Bolduc et al., 2012), and the localized expression of *rs1* predicted and subtended the initiation of axillary meristems on the flanks of the IM (Schneeberger et al., 1995). Interestingly, the *S. viridis* homologs of the BP interaction partner, *BRAHMA* (*BRM*), and a closely related gene, *SPLAYED* (*SYD*), both encoding SWI/SNF chromatin remodeling ATPases in Arabidopsis (Wagner and Meyerowitz, 2002; Zhao et al., 2015), Sevir.2G237500 and Sevir.4G112300, respectively, were also found cluster 11 (**Supplementary Table S3**). In Arabidopsis, *BRM* upregulates *CUC* genes (Kwon et al., 2006), which are essential players in lateral meristem establishment and proper boundary formation; *SvCuc2* (Sevir.4G124400) and *SvCuc3* (Sevir.6G213600) were co-expressed in cluster 22. In addition, two class II TCP TFs orthologous to *branch angle defective 1* (*bad1*)/*wavy auricle blade 1* (*wab1*) (Bai et al., 2012; Lewis et al., 2014) and *teosinte branched 1* (*tb1*) (Doebley et al., 1997) were co-expressed in cluster 22 [*SvBad1* (Sevir.2G210100) and *SvTb1* (Sevir.9G122200), respectively]. Consistent with their expression profiles during *S. viridis* inflorescence development (**Figure 2**), their localized expression overlaps in axillary meristems of young maize tassels and both genes contribute to the regulation of branch outgrowth (Bai et al., 2012).

### Group 4: Floral Organ Development

Genes in Group 4 (clusters 1, 6, 14, 16, 17, and 23) progressively increased in expression after 14 DAS and peaked largely at 18 DAS, coinciding with floral organ formation and differentiation (**Figure 3A** and **Supplementary Figure S6**). A number of genes encoding MIKC-type MADS-box TFs were found in this group, which was expected given their conserved roles as master regulators of floral organ identity across species (Theißen et al., 2016; **Figures 2** and **Supplementary Figure S5**). Among them were two B-class function TFs known to regulate lodicule identity and stamen development and co-expressed in cluster 6; an ortholog of the maize *silky1 (si1)* gene (*SvSi1*; Sevir.4G289400) and homolog of *OsMADS4* from rice (*SvMADS4*; Sevir.3G242100) (Ambrose et al., 2000; Nagasawa et al., 2003; Yao et al., 2008). A number of C-class, *AGAMOUS*-like genes were also co-expressed in cluster 6, including *SvBde1* (Sevir.1G278500), orthologous to the maize *bearded ear 1* (*bde1*) gene (Thompson et al., 2009), and homologs of rice *OsMADS17*, *OsMADS3*, and *OsMADS58*, Sevir.7G221900, Sevir.5G141300, and Sevir.3G074600, respectively (Yamaguchi et al., 2006). Also, three *SEPALLATA*-like E-class genes were expressed in cluster 6 including homologs of rice *OsMADS1*/*LEAFY HULL STERILE 1* (*LHS1*), *OsMADS7*, and *OsMADS8*, Sevir.9G494300, Sevir.6G230500, and Sevir.2G277000, respectively, which all presumably function in specifying floral organ identity (Jeon, 2000; Zahn et al., 2005; Cui et al., 2010).

Additional genes that function in floral organ specification were also found in Group 4. Among these, *SvTga1* (Sevir.6G230400), the ortholog of *teosinte glume architecture 1* (*tga1)* encoding an SBP TF that regulates glume development and was a key domestication locus in maize (Dorweiler and Doebley, 1997; Preston et al., 2012), was found in cluster 16 (**Figure 3B** and **Supplementary Figure S3**). In addition, orthologs of the C2H2 TF *STAMENLESS 1* from rice (Sevir.5G086100; *SvSl1*) (Xiao et al., 2009) and maize *yabby 15*/rice *TONGARI-BOUSHI 1* (*TOB1*) (Sevir.1G255900; *SvTob1*) (Tanaka et al., 2012), were expressed in clusters 6 and 14, respectively (Tanaka et al., 2012). *TOB1* and close homologs regulate maintenance and fate of reproductive meristems whereas *OsSL1* regulates floral organ identity. Sex determination genes from maize were also associated with Group 4, including orthologs of maize *anther ear 1* (*an1*), a terpenoid synthase involved in the gibberellic acid biosynthesis (Sevir.9G265300*; SvAn1*) (Bensen et al., 1995) and *tassel seed 2* (*ts2*) (Sevir.9G439800; *SvTs2*) (DeLong et al., 1993) in cluster 17 and 16, respectively (**Figure 3A** and **Supplementary Table S3**).

## Co-expression Network Analysis Using WGCNA

To extend our co-expression analyses, we also performed a WGCNA (Langfelder and Horvath, 2008). We used WGCNA to independently define modules of co-expressed genes and to construct a co-expression network for early *S. viridis* inflorescence development (**Supplementary Table S6**). WGCNA produced 14 co-expression modules based on parameters detailed in the Section “Materials and Methods,” which were highly consistent with our FCM results; i.e., each WGCNA module largely corresponded to the sum of several FCM clusters (**Supplementary Figure S8**). In addition to grouping genes into co-expression modules, WGCNA was used to determine the interconnectedness of genes within and between modules. A strength value (i.e., weight) was assigned to the connection (edge) between each gene (node) and every other gene in the network. An integrated co-expression network was generated after filtering gene-to-gene connections with very low connectivity (weight >0.02; available in NCBI GEO).

**FIGURE 5:**
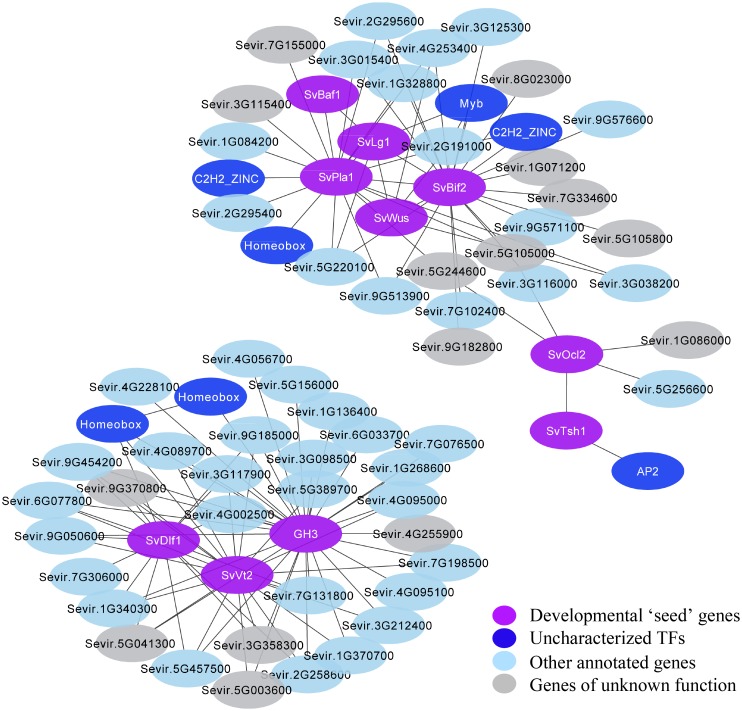
Two sub-networks extracted from the WGCNA were defined based on gene connectivity to a set of co-expressed regulators of inflorescence development (shown in *purple*). Genes (or nodes) were included if they were connected to these developmental genes by edges with a weight >0.2. Developmental regulators within these sub-networks either show high connectivity (e.g., *SvVt2*, *SvBif2*) or low connectivity (e.g., *SvTsh1*, *SvBaf1*). Uncharacterized TFs are indicated in *dark blue*, other genes with assigned functional annotations in *light blue*, and genes of unknown function in gray.

To define sub-networks related to specific developmental processes and potentially identify new regulatory factors, we filtered the co-expression network for genes with strong connections to known genes of interest within a module. For example, we explored a co-expression module that included *SvLg1*, *SvBaf1*, and *SvBif2*, which are known players in boundary formation and axillary meristem initiation (“Plum” module; **Supplementary Figure S8** and **Supplementary Table S6**). To reduce complexity in visualization and interpretation of this module, we included only genes with strong interactions (edges between genes having a weight >0.2) to a set of ten predicted developmental genes (**Figure 5** and **Supplementary Table S7**). One sub-network was defined that included strong co-expression “interactions” among *SvDfl1* (Sevir.2G302300), a homolog of maize *delayed flower 1* (Muszynski et al., 2006), *SvVt2* and a GH3 IAA-amido synthase (Sevir.9G364900) (**Supplementary Figure S7**), homologous to Arabidopsis *YADOKARI 1* (*YDK1*) and predicted to regulate auxin levels (Staswick et al., 2005), via a common set of genes. In another sub-network, *SvPla1* (Sevir.9G259300), a cytochrome P450 homologous to Arabidopsis *PLASTOCHRON1* (Miyoshi et al., 2004; Sun et al., 2017), was identified as a hub gene directly or indirectly connected to *SvBaf1*, *SvLg1*, and *SvBif2*, consistent with its conserved role in lateral organ initiation (**Figure 5** and **Supplementary Table S7**). Within these subnetworks, some developmental regulators showed high connectivity to many other genes, while others showed relatively low connectivity (**Figure 5**).

## *Setaria viridis* as a Comparative Model for Panicoid Grass Inflorescence Development

Our transcriptomics analysis in *S. viridis* identified homologs of known developmental regulators as well as uncharacterized genes with putative roles in panicoid grass inflorescence development. Based on spatiotemporal expression profiles, we showed that genes controlling transitions in meristem types during inflorescence development are largely conserved between *S. viridis* and maize (**Figure 4**). Since the diversity of inflorescence architectures found across grasses are largely determined by subtle regulatory variation on common developmental processes, it is not surprising that suites of genes have conserved functions across grasses, and often analogous functions in more distantly related species. For example, *tsh1/NL1* genes share conserved functions in boundary establishment and bract suppression in maize and rice (Wang et al., 2009; Whipple et al., 2010), whereas the close Arabidopsis homolog, *HANABA TARANU*, functions in floral organ development (Zhao et al., 2004). However, in some cases orthologous genes can take on context-specific functional roles. The rice ortholog of *baf1*, *DEPRESSED PALEA 1* (*DP1*), regulates palea formation, and floral organ number (Jin et al., 2011), while its close homolog in Arabidopsis, *AHL22*, functions in the floral transition (Yun et al., 2012).

We anticipate that species-specific expression differences underlie the variation in inflorescence form in *Setaria* spp. compared to maize and other closely related grasses. While we are beginning to elucidate some of the genes responsible for these morphological differences through mutant screens and experimental analyses (Yang et al., 2017), identifying subtle variation in gene regulation at the network level will require parallel transcriptomics resources from other species. In addition, using co-expression analyses to prioritize novel candidate genes as having previously undefined functions in development will also be most powerful when evaluated in the context of comparable datasets. Thus, a detailed knowledgebase of how the expression of individual genes shift during development and how co-expressed gene modules are rewired across grass species, is invaluable to understanding the genetic basis for morphological diversity in grass inflorescence architecture.

The apparent similarity between maize and *S. viridis* at the transcriptome level supports its use as a functional model for panicoid cereals, especially when paired with rapid advances in gene editing and transformation technologies (Zhu et al., 2017). Our transcriptomics analyses provide a platform for gene discovery in *S. viridis* inflorescence development and a comparative model for studying diverse architectures of agronomically important cereal crops. With respect to the former, it is anticipated that these data will help uncover novel expression patterns associated with the unique features of *S. viridis* inflorescence morphology, which can be translated to other millets (Huang et al., 2016), including subsistence crops in many developing countries that remain largely untapped for genetic improvement.

## Accession Numbers

RNA-seq data (raw sequence reads and processed data files) generated in this study and the WGCNA gene co-expression matrix are available from the NCBI Gene Expression Omnibus (GEO; www.ncbi.nlm.nih.gov/geo/) under accession number GSE118673.

## Author Contributions

AE and EK conceptualized and supervised the work. MB established the developmental sampling scheme. JY and MB performed SEM and RNA-seq experiments. JY and CZ performed bioinformatics analyses and data visualization. CZ, JY, EK, and AE wrote the paper. All authors read and approved the final manuscript.

## Conflict of Interest Statement

The authors declare that the research was conducted in the absence of any commercial or financial relationships that could be construed as a potential conflict of interest.

## Footnotes

1 phytozome.jgi.doe.gov

2 http://mev.tm4.org/

3 http://plants.ensembl.org

4 https://blast.ncbi.nlm.nih.gov/

5 https://www.maizegdb.org/assembly

6 hmmer.org

7 http://www.cytoscape.org

## Supplementary Material

FIGURE S1 Correlations of global gene expression profiles across all samples.

FIGURE S2 Expression profiles of orthologs of known developmental genes across six stages of *Setaria viridis* inflorescence development showing standard deviation among biological replicates.

FIGURE S3 Dynamic expression profiles of TF families during inflorescence development in *Setaria viridis*: LOB, NAC, AP2/ERF, C2H2, bZIP, and bHLH families.

FIGURE S4 Expression profiles of uncharacterized TFs with highly dynamic changes during inflorescence development.

FIGURE S5 Phylogenetic relationship of *Setaria viridis* MIKC-type MADS-box TFs with homologous genes in rice, maize, and Arabidopsis.

FIGURE S6 Cluster number selection for FCM and developmental trajectories of 25 clusters.

FIGURE S7 Expression profiles of auxin-related genes from Groups 2 and 3.

FIGURE S8 Assignment of genes to co-expression modules using WGCNA and comparison to FCM analyses.

TABLE S1 RNA-seq library sequencing and mapping statistics.

TABLE S2 Gene expression values for all *S. viridis* genes, their closest orthologs in other species, and available functional annotations.

TABLE S3 Cluster assignment of 11,425 dynamically expressed genes and associated functional annotation.

TABLE S4 Expression values and annotations of selected transcriptional factor (TF) families.

TABLE S5 Expression values and functional annotations of auxin-related genes from Groups 2 and 3.

TABLE S6 Output from WGCNA using 11,425 dynamically expressed genes showing the relative connectivity of each gene to each of 14 modules.

TABLE S7 Genes (or nodes) and their connections (edges) to each other within the two sub-networks depicted in **Figure 5**.
