# Supplementary material for: A Dynamic Co-expression Map of Early Inflorescence Development in Setaria viridis Provides a Resource for Gene Discovery and Comparative Genomics
Source: Front Plant Sci. 2018 Sep 12;9:1309. doi: 10.3389/fpls.2018.01309 (PMC6143762; doi:10.3389/fpls.2018.01309)
Supplement: FIGURE S1 — Correlations of global gene expression profiles across all samples. [file Presentation_1.PDF]

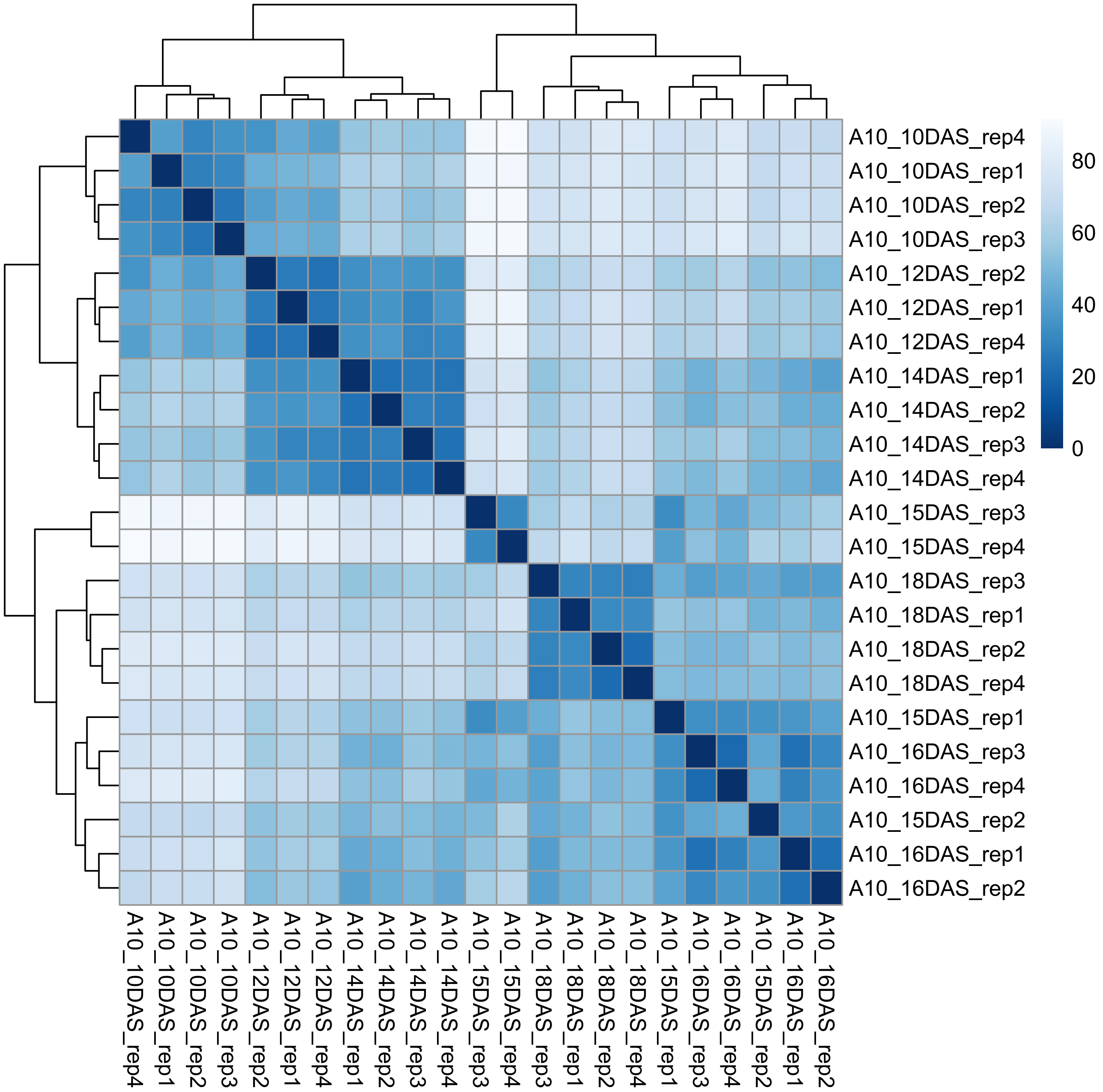

Figure S1. **Correlations of global gene expression profiles across all samples**: biological replicates and developmental stages. TPM values for 35,214 genes were used to determine correlations among samples. In general, biological replicates representing a given developmental stage clustered together in a distinct group. Two 15 DAS samples clustered with 16 DAS samples, which was not surprising given the similarity in stages and complexity of the pooled inflorescence primordia at this time. Surveying expression of individual developmental genes and TFs in Figures S2 and S4, respectively, indicated that differences between 15 and 16 DAS can be resolved and supported by Standard Deviations among biological replicates. Expression levels of individual replicates are provided for each gene in Supplementary Table S2.

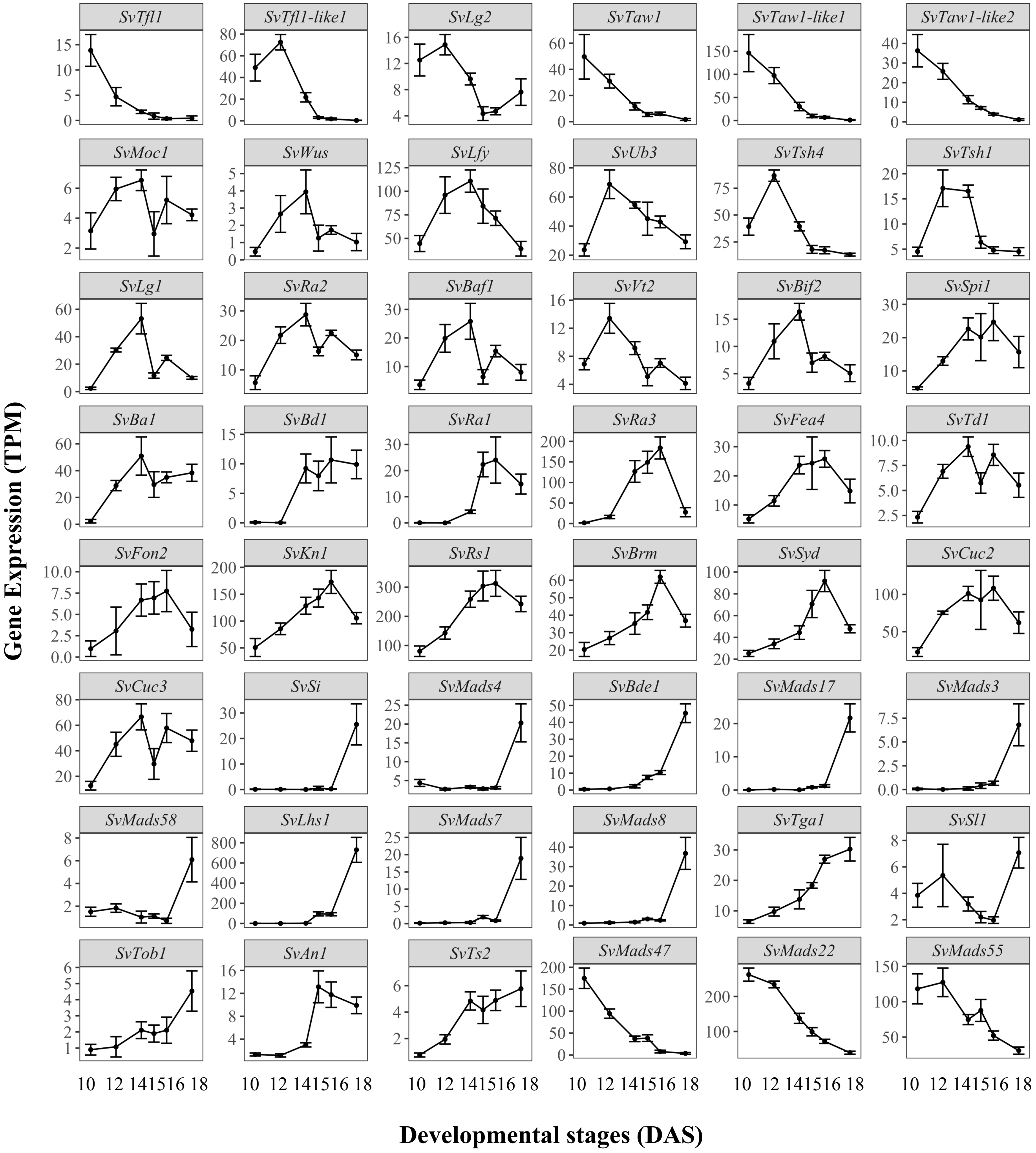

Figure S2. Expression profiles of orthologs of known developmental genes across six stages of *S. viridis* inflorescence development showing standard deviation among biological replicates. These profiles support the robustness of the RNA-seq data across development and biological replicates; dynamic changes across development are consistent with what has been shown for orthologs of these genes in other species and variation among replicates is low and not biased for a specific stage.

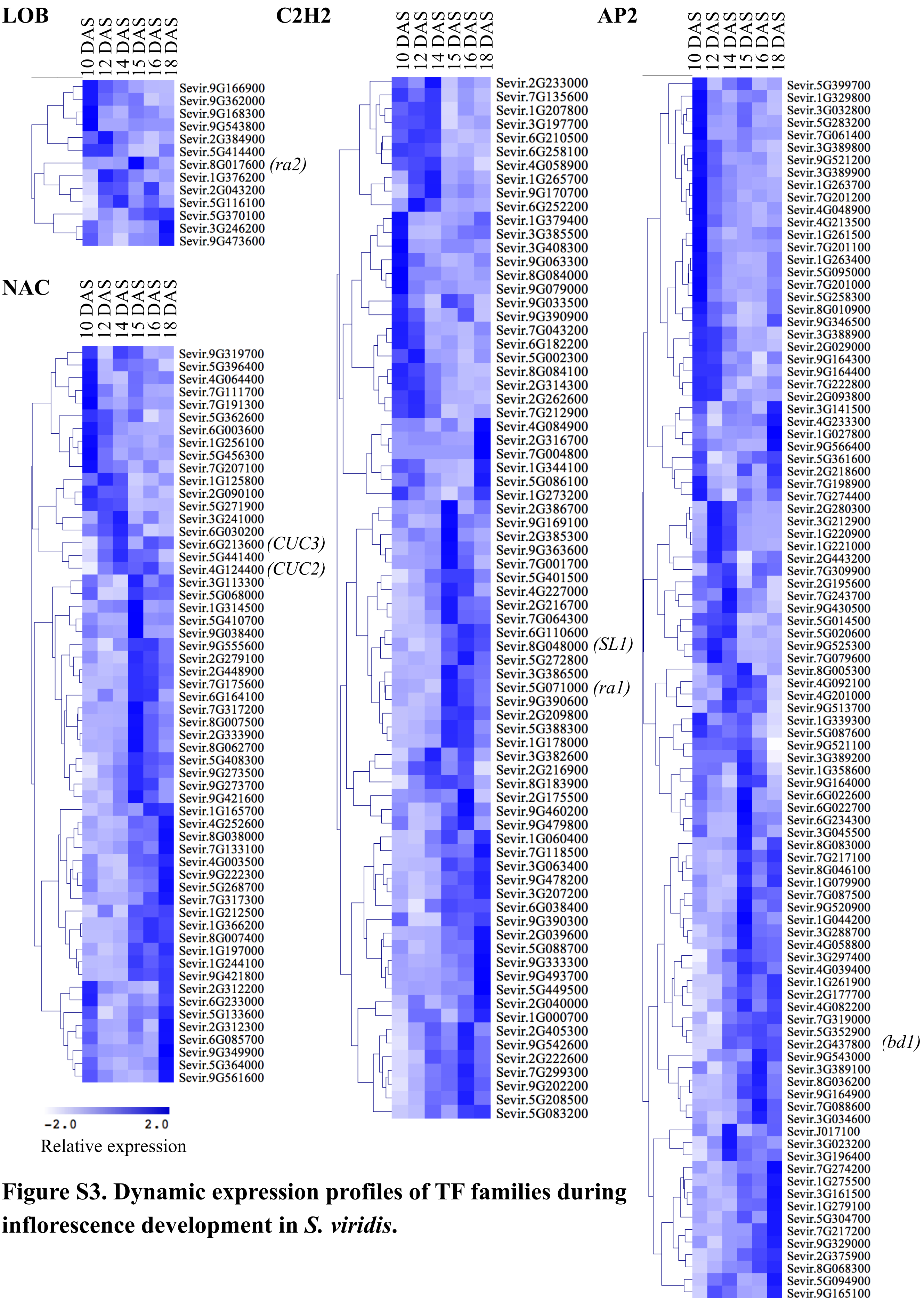

Figure S3. Dynamic expression profiles of TF families during inflorescence development in *S. viridis*.

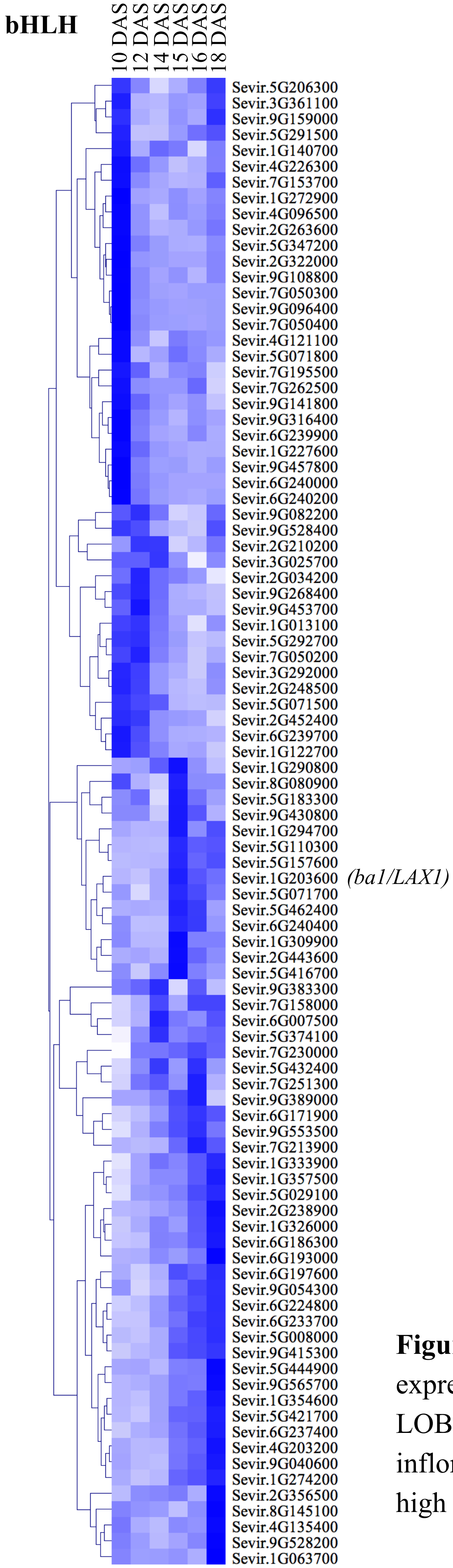

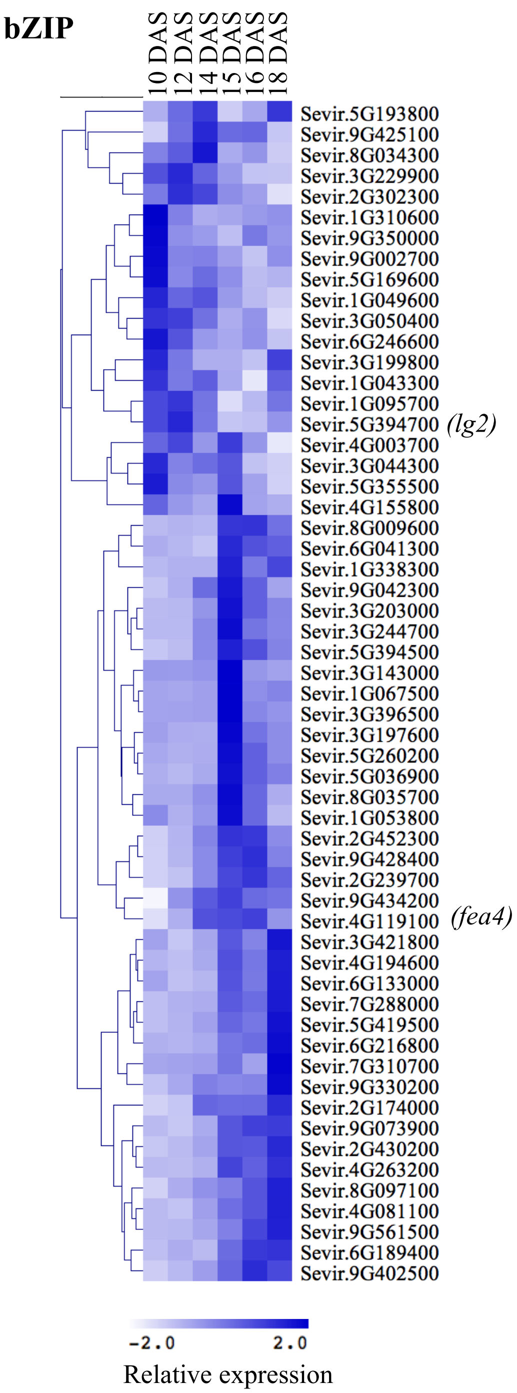

Figure S3 continued. Heatmaps show normalized expression patterns of individual members of TF families: LOB, NAC, C2H2 zinc finger, AP2, bHLH, and bZIP during inflorescence development. White-to-dark blue means low to high expression.

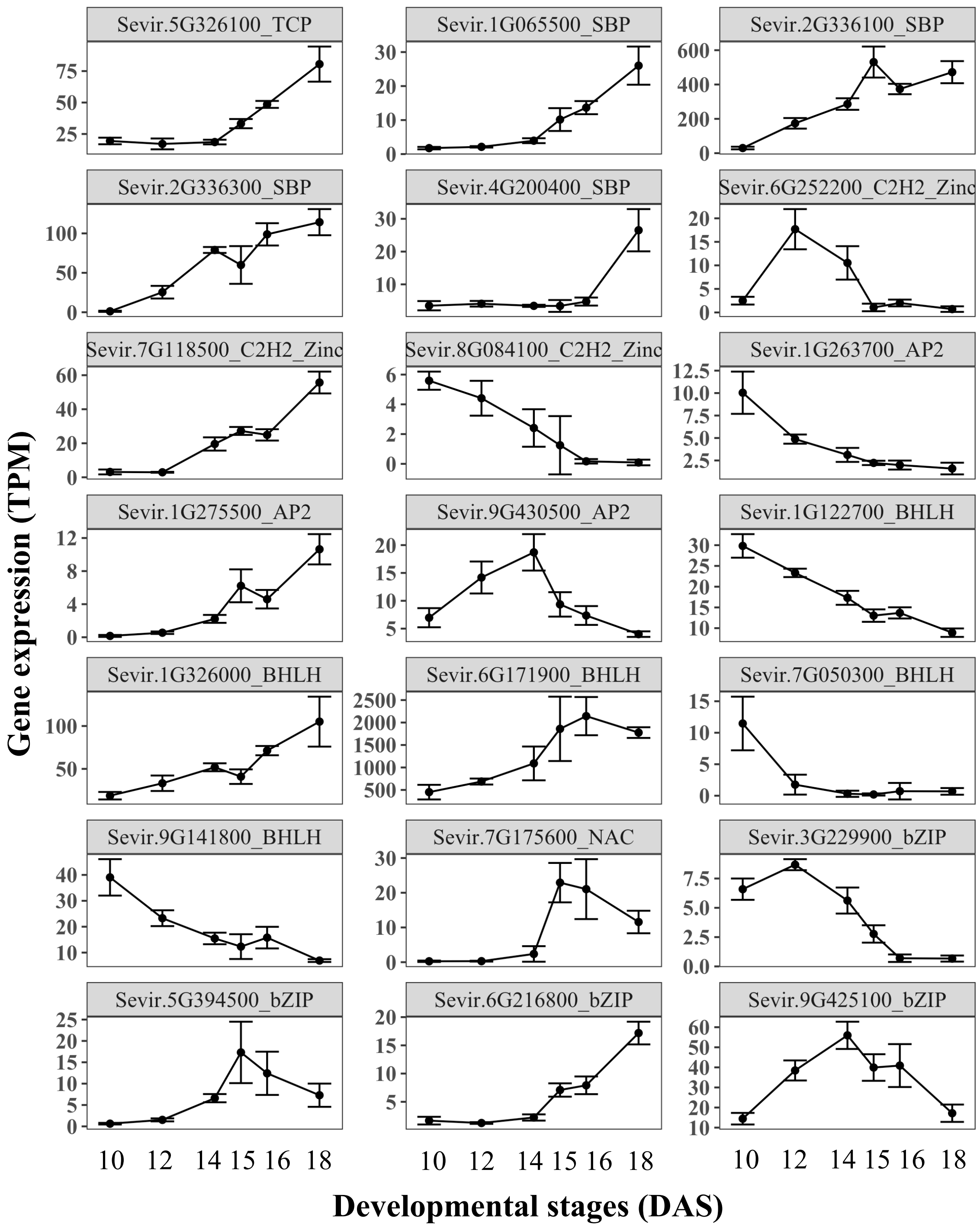

Figure S4. Expression profiles of uncharacterized TFs with highly dynamic changes during inflorescence development. Previously uncharacterized TFs from diverse families were identified as promising candidates in regulation of inflorescence development based on their expression profiles: all demonstrated large fold changes and high expression levels with a collective TPM > 10 across the six stages.

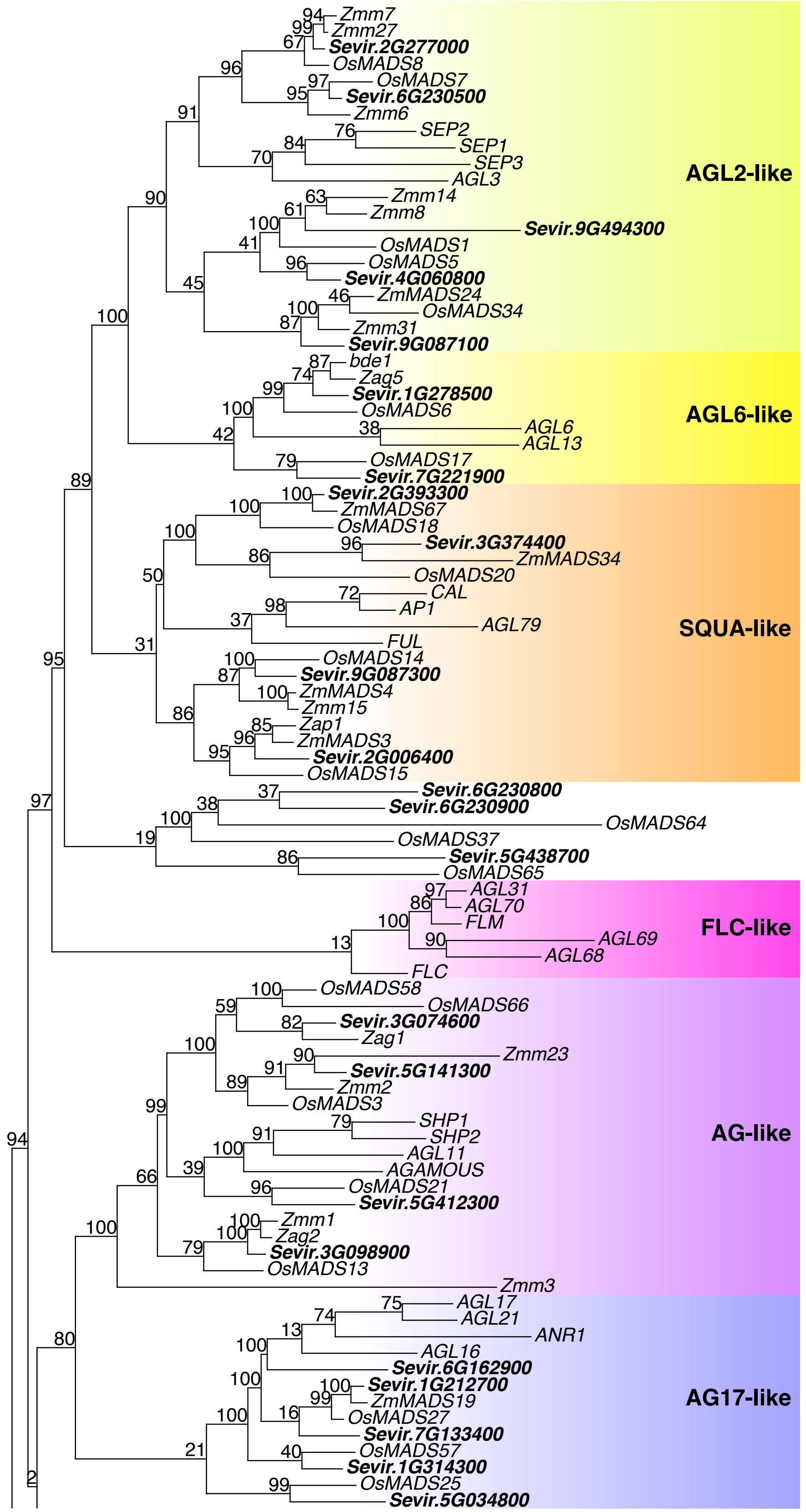

Figure S5. Phylogenetic relationship of *S. viridis* MIKC-type MADS-box TFs with homologous genes in rice, maize, and Arabidopsis.

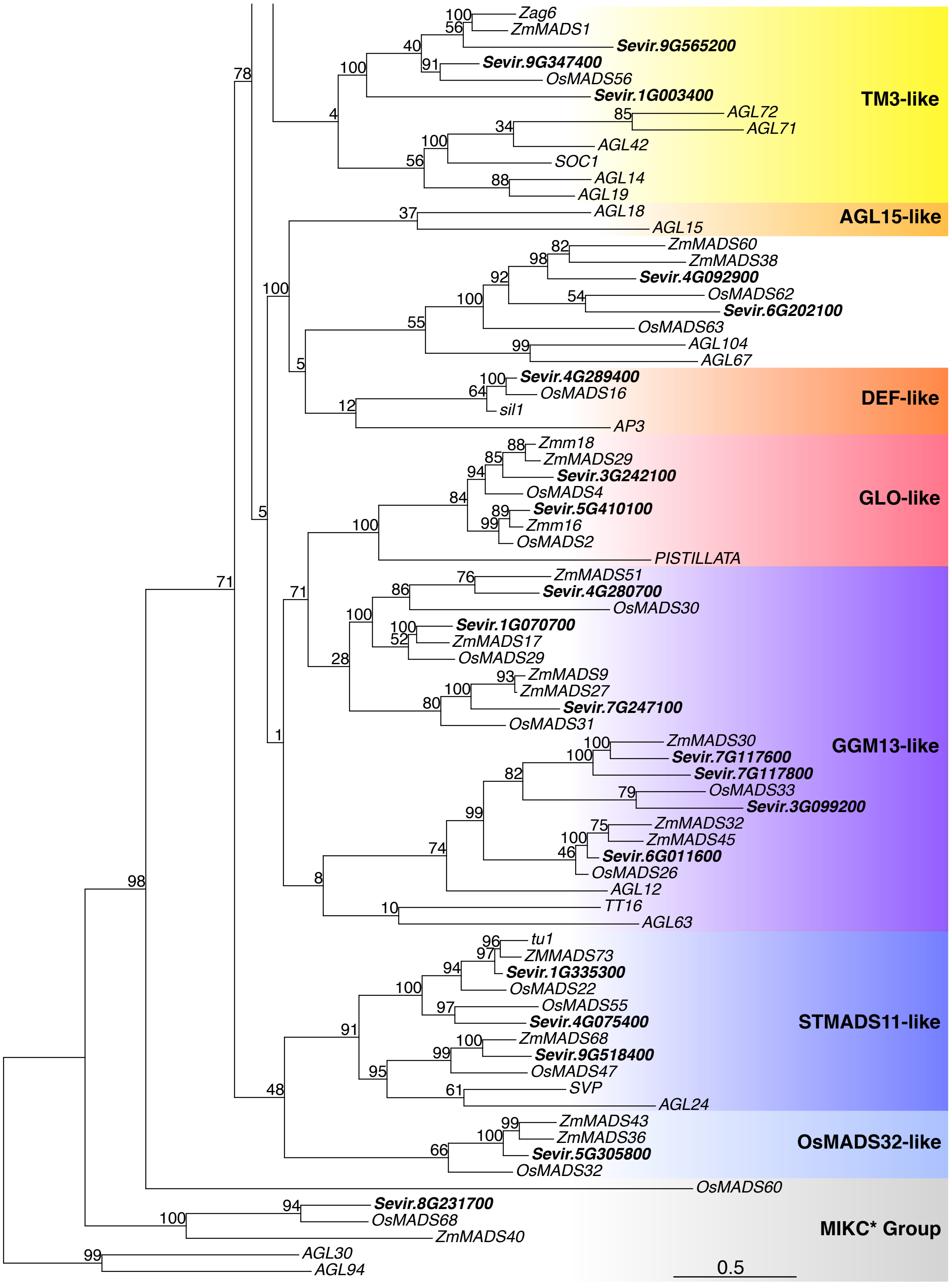

Figure S5 continued. The tree was constructed using protein coding sequences from maize, rice, Arabidopsis and S. *viridis* and a maximum likelihood method by RAxML. Colors depict previously annotated MADS-box clades. *S. viridis* MIKC-type MADS-box genes are highlighted in bold.

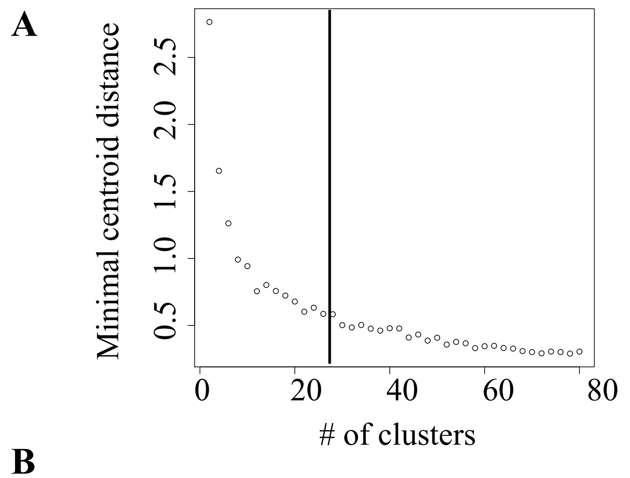

Figure S6. Cluster number selection for FCM and developmental trajectories of 25 clusters. (**A**) The Dmin plot used to determine the optimal number of clusters shows the relationship between the minimal distance among cluster centers (centroids) with cluster number. (**B**) 25 clusters generated from FCM analysis.

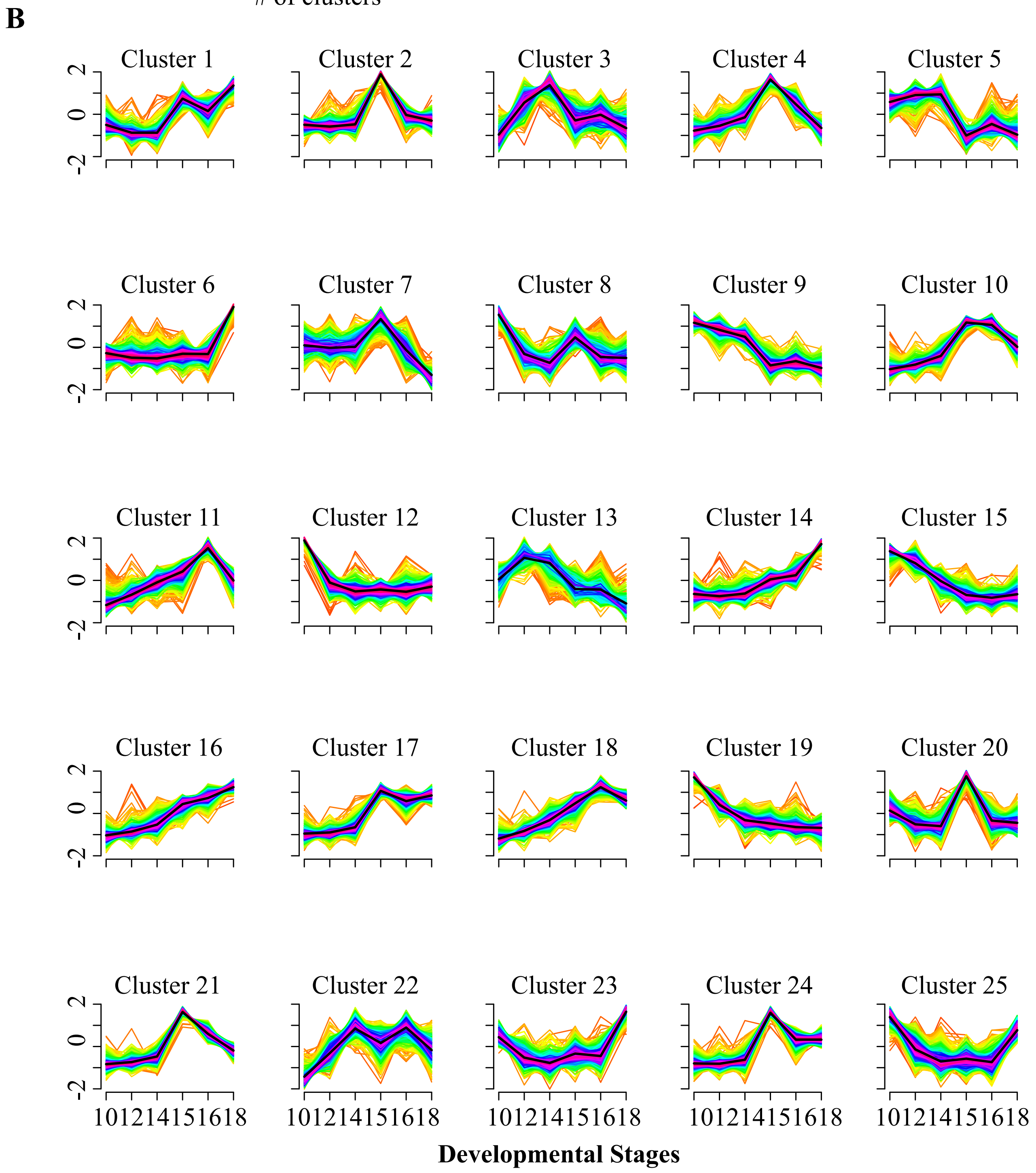

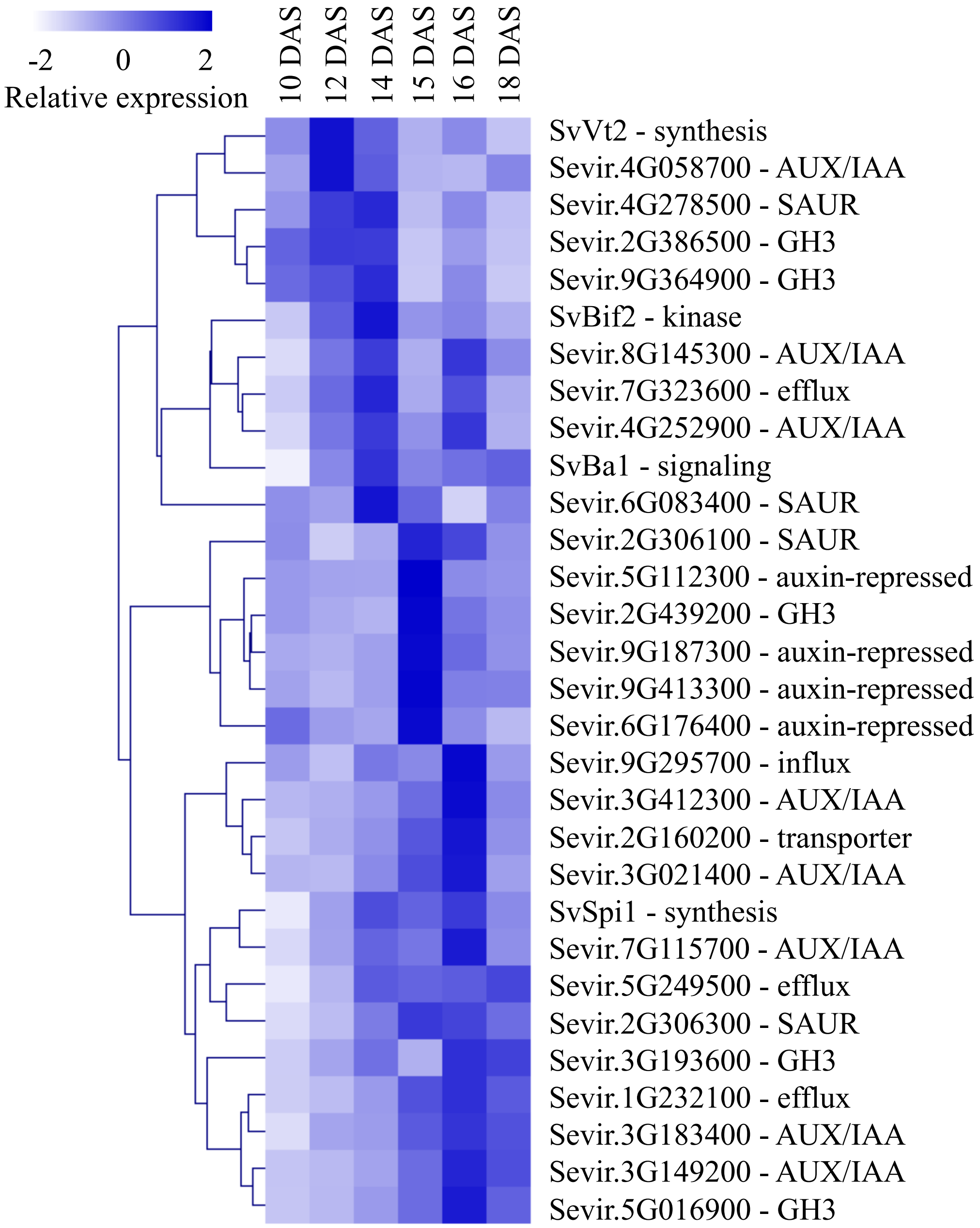

Figure S7. Expression profiles of auxin-related genes from Groups 2 and 3. Auxin-related genes include those involved in the synthesis, transport, signaling and response to auxin. Functional classifications listed to the right of the *S. viridis* gene names are based on annotations of homologous genes extracted from Phytozome, which are listed in Supplemental Table S5. White-to-dark blue = low-to-high gene expression. Only genes with a collective expression level of >10 TPM across the six stages are shown.

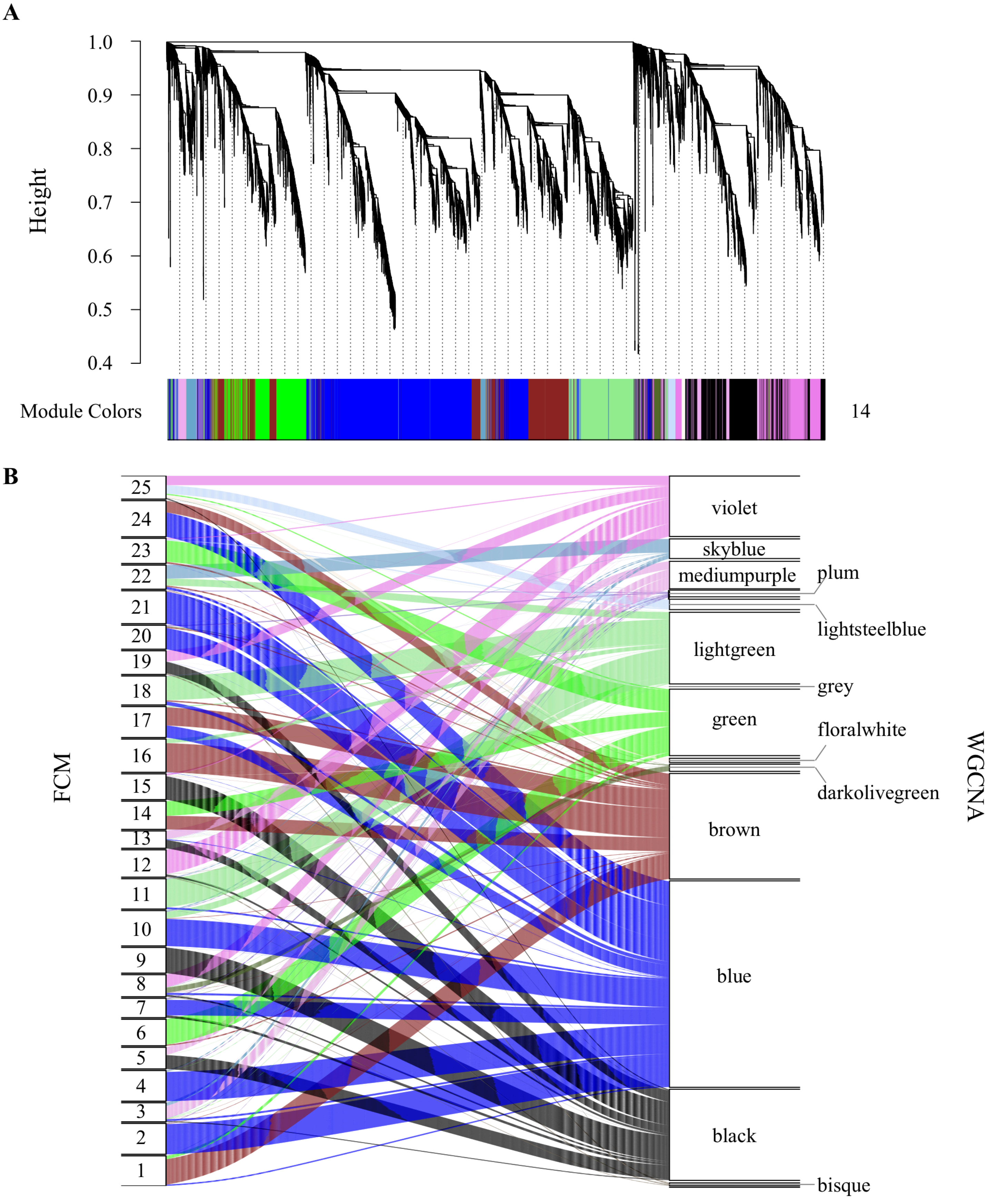

Figure S8. Assignment of genes to co-expression modules using WGCNA and comparison to FCM analyses. (A) The cluster dendrogram shows hierarchical clustering of 11,425 genes and their assignments to 14 modules of co-expressed genes by a major tree branch. **(B)** The alluvial diagram shows association of these 14 modules with 25 co-expression clusters by FCM. Each line represents a gene and the color indicates the module it associates with WGCNA on the right. Cluster numbers from FCM analysis are shown on the left.
